# A nutrition environment measures survey for dollar stores (nems-ds) in rural south carolina: assessing nutrition equity and WIC readiness

**DOI:** 10.1186/s12889-026-27446-6

**Published:** 2026-04-29

**Authors:** Hadis Dastgerdizad, Theresa Andrasfay, Lovelyn Adaobi Isiani

**Affiliations:** 1 Department of Sociology, Global Health Studies, University of Miami, 5202 University Drive, 120-G Merrick Building, Coral Gables, FL 33146 USA; 2 Department of Public Health, California State University San Marcos, San Marcos, USA; 3 Department of Sociology and Criminology, University of Miami, Coral Gables, FL 33146 USA

**Keywords:** WIC readiness, Rural food environment, Area deprivation index, Nutrition equity, Nutrition environment measures survey (NEMS-DS), Dollar stores

## Abstract

**Background:**

Rural areas in the southern United States experience a disproportionate burden of diet-related disorders, driven by limited access to nutritious and affordable foods. Dollar stores have rapidly grown in these regions, including South Carolina’s Lowcountry, where they often replace traditional grocery stores and shape residents’ nutritional behaviors. However, their food environment and readiness to serve as vendors for the Women, Infants, and Children (WIC) nutrition program remain under-examined. This study addressed this gap using a newly developed Nutrition Environment Measures Survey for Dollar Stores (NEMS-DS), which captures dimensions overlooked by previous assessment tools, particularly given dollar stores’ reliance on shelf-stable and ultra-processed foods.

**Methods:**

A quantitative, cross-sectional study was conducted in 13 dollar stores across six rural counties in the Lowcountry. All Lowcountry stores were located in rural areas, and the majority (46%) were in high-deprivation areas identified through the Area Deprivation Index. Food environments of sampled stores were assessed across three domains: (1) healthy food availability; (2) WIC readiness; and (3) marketing of healthy versus unhealthy items (e.g., shelf-space allocation). Descriptive statistics, F-tests, and chi-square tests were employed to compare Lowcountry stores with 202 dollar stores from a parallel multiple state assessment.

**Results:**

Lowcountry stores demonstrated limited healthy food availability scores (mean 23 out of a possible 62) and low WIC readiness (mean 51 out of a possible 190). Stores allocated only 12% of the total shelf space to healthy items. None marketed healthy foods at checkout, whereas 100% promoted sugar-sweetened beverages and ultra-processed snacks, patterns consistent with stores in comparison research sites. Although fresh produce availability was slightly higher in the Lowcountry than in the comparison sample, frozen and shelf-stable produce availability was significantly lower.

**Conclusion:**

Dollar stores in the Lowcountry mirrored national patterns characterized by heavy reliance on ultra-processed foods in rural high-poverty settings. Findings inform policy discussions regarding WIC vendor eligibility, retail product placement practices, and marketing regulation to improve nutritional behaviors, promote equitable access to nutritious foods, and prevent diet-related chronic disease in rural communities.

**Supplementary Information:**

The online version contains supplementary material available at 10.1186/s12889-026-27446-6.

## Introduction

Nutrition-related chronic disorders, including type II diabetes, obesity, hypertension, and cardiovascular disease, remain leading causes of preventable morbidity and mortality in the United States (U.S.) [1–5]. These burdens disproportionally affect low-income, rural, and racially diverse communities, that experience limited access to affordable and nutritious foods [6–13]. South Carolina (SC), with a significant proportion of its land classified as rural, exemplifies these disparities, with adult obesity (36%) and diabetes (13.3%) prevalence exceeding national averages [14–22]. Such disparities underscore the need to move beyond individual behavior change and to investigate structural and environmental inequities [6, 8, 10, 23].

Understanding the characteristics of the community food environments is central to improving dietary patterns and reducing chronic disease disparities among rural communities [8, 23]. Dollar stores have expanded rapidly across the rural South, serving as primary food retail outlets in many rural areas [24–34]. Dollar stores significantly influence dietary choices and overall nutritional behaviors in rural populations [34–42], yet they typically offer shelf-stable and ultra-processed foods with minimal fresh options [37, 41–43]. In many rural areas, the entry of dollar stores often leads to the closure of local grocery stores, further reducing access to healthier food options and aggravating food insecurity [44–47].

In the Lowcountry, high food insecurity and historical marginalization reflect deep structural inequities [20, 48–50]. These inequities are further compounded by recent Supplemental Nutrition Assistance Program (SNAP) benefit reductions, which may reduce support for low-income rural families and increase reliance on the Women, Infants, and Children (WIC) program [51, 52]. Despite dollar stores' ubiquity in rural food landscapes and their acceptance of SNAP, they are generally ineligible to serve as WIC vendors because WIC-approved products are not widely available. To be authorized as WIC vendors, stores must meet federal minimum stocking requirements across multiple food categories, including fruits and vegetables, whole grains, dairy products, proteins, infant foods and formula, with specific requirements for variety, package size, and nutritional composition [53]. This presents a critical but understudied gap as expanding WIC authorization to dollar stores has the potential to increase access to healthy foods for low-income mothers and children in rural areas; however, little is known about dollar stores' WIC-readiness.

The Nutrition Environment Measures Survey (NEMS) is a reliable and validated instrument that has played an essential role in advancing research on food environments improvement by enabling examination of relationships between healthy food availability and factors, such as nutritional behaviors, health outcomes, and intervention-driven changes in food retail settings [54, 55]. However, as retail formats vary, NEMS measures require ongoing adaptation to remain applicable across diverse food contexts [54, 55]. The original NEMS for Stores (NEMS-S), which were developed for large supermarkets or NEMS for Corner Stores (NEMS-CS) for smaller food retailers, are not well-suited to assess dollar stores' food environments [54–56]. These tools also do not assess dollar stores' capacity to meet WIC program standards. Therefore, the Nutrition Environment Measures Survey for Dollar Stores (NEMS-DS) tool was developed by a multidisciplinary research team from the Dollar Store Research Work Group. This work group is part of a larger Healthy Food Retail (HFR) Work Group, supported by Healthy Eating Research (HER) a program of the Robert Wood Johnson Foundation (RWJF), and the Nutrition and Obesity Research and Evaluation Network (NOPREN) (funded by the Centers for Disease Control and Prevention (CDC)).This research workgroup included 11 researchers spanning multiple public health disciplines and U.S. institutions with experience and expertise in implementing and evaluating healthy food retail interventions. The newly developed NEMS-DS addresses prior limitations by providing a store-format-specific tool designed to assess the availability, visibility, shelf space, and marketing of healthy and unhealthy foods in dollar stores, as well as their readiness to become WIC vendors [54–56].

This study is grounded in the Community Nutrition Environment (CNE) and Social-Ecological Models (SEM), which highlight how structural, community, and food-environment factors shape consumer purchasing patterns and dietary behavior [57, 58]. Applying these frameworks allows for an integrated assessment of environmental influences from limited healthy food placement to inadequate WIC-eligible stock that constrain consumer choice and reinforce rural nutrition inequities.

No prior work has jointly assessed the nutrition environment and WIC readiness of rural Lowcountry dollar stores using a format-specific standardized tool, nor compared findings across states using standardized metrics. This study represents one of the first multi-state applications of NEMS-DS within dollar store settings across varied geographic contexts. Filling this gap is critical for informing policies to improve healthy food access in underserved rural communities. This study aimed to 1) assess the availability, visibility, marketing, and shelf-space allocation of healthy versus unhealthy foods in 13 rural dollar stores in the Lowcountry using the NEMS-DS tool; 2) evaluate the WIC readiness of these stores; and 3) compare findings from rural Lowcountry stores to those from 202 dollar stores across multiple states, situating local conditions within broader national patterns. By generating this evidence, this study contributes to the literature on rural food environments and informs policy discussions aimed at improving equitable food access in underserved communities.

## Methods

### Study design & setting

We conducted a quantitative cross-sectional assessment of the nutrition environments of 13 dollar stores located in six rural counties of the Lowcountry, including Allendale, Beaufort, Charleston, Colleton, Hampton, and Jasper counties (Table 1). The Lowcountry sampled dollar stores represent a subset of a larger multiple state parallel study of dollar stores across 10 U.S. states (n = 13stores in the Lowcountry, SC in addition to 202 stores in other sampled states). These 10 states included New York, Texas, Illinois, Florida, Maryland, Georgia, Louisiana, Michigan, Southern California, and South Carolina.

**Table 1:** Store-level and neighborhood characteristics by research site

	**South Carolina (Lowcountry)** **(n = 13)**		**Other Research Sites** **(n = 202)**		
	N (%) or Mean, Standard Deviation (SD)		N (%) or Mean (SD)		*p*-value
County					
Hampton	3	(23%)			
Jasper	3	(23%)			
Allendale	2	(15%)			
Charleston	2	(15%)			
Colleton	2	(15%)			
Beaufort	1	(8%)			
Store Brand					0.06
Dollar Tree	2	(15%)	83	(41%)	
Dollar General	8	(62%)	63	(31%)	
Family Dollar	3	(23%)	56	(27%)	
ADI level^a^					0.07
Low	5	(38%)	29	(14%)	
Medium	2	(15%)	50	(25%)	
High	6	(46%)	120	(60%)	
Urban Status					< 0.001
Urban/suburban	0	(0%)	181	(91%)	
Rural	13	(100%)	18	(9%)	
Percent non-Hispanic White	47	(20)	38	(33)	0.33
Percent non-Hispanic Black	41	(17)	28	(34)	0.15
Percent Hispanic	7.8	(8.4)	27	(31)	0.03
Percent non-Hispanic other races	3.3	(2.0)	6.7	(8.3)	0.14
Accept SNAP					0.72
No	0	(0%)	13	(6%)	
Yes	13	(100%)	188	(94%)	
Number of checkouts	2.2	(0.8)	3.4	(1.2)	< 0.001

WIC readiness scores were low across all stores (Table 2). Lowcountry stores had an average score of 51 out of a potential maximum of 190, similar to other research sites

^a^ADI level is based on the state deciles of the Area Deprivation Index (ADI). Deciles 1–3 are categorized as low ADI, 4–6 as medium ADI, and 7–10 as high ADI

*p*-values are from chi-square tests for categorical variables and one-way ANOVA (F-tests) for continuous variables comparing stores in South Carolina to stores in other research sites

Dollar stores were identified through publicly available sources and verified through online searches, telephone calls, or in-person visits. For each site, stores were matched to state-level deciles of the Area Deprivation Index (ADI) and categorized as low (1–3), medium (4–6), or high (7–10) deprivation. The ADI is a composite measure of 17 socioeconomic indicators reflecting income, employment, education, housing, and household characteristics [59–62].

### Sampling strategy

At each research site, including the Lowcountry, stores were first stratified by ADI level. Because it is relatively uncommon for dollar stores to be located in low-deprivation areas, any stores located in low-ADI communities were included in the sample. The remaining stores were randomly sampled from the medium- and high-ADI strata until the research site's target sample size was reached or all eligible stores were included. We employed a stratified random sampling approach across ADI levels in the Lowcountry, with intentional prioritization of rural and high-poverty communities to ensure adequate representation of areas with greater structural barriers to healthy food access [59]. From the 28 national chain dollar stores (Dollar General, Family Dollar, Dollar Tree) identified in the Lowcountry, 13 were eligible and accessible for assessment. Stores not included were permanently closed (n = 9), or temporarily inaccessible (n = 6).

### Data collection

Quantitative data was collected using NEMS-DS. The NEMS-DS tool was developed in three phases: development of the NEMS-DS draft, pilot testing of the draft tool, tool revision and finalization. The NEMS-DS was developed based on multiple prior food environment assessment tools designed for grocery stores, such as (NEMS-S), NEMS-CS, and the Market Basket Assessment Tool (MBAT) as well as institution-level tools used for previous dollar store assessments, such as the tool used in a study of small food retailers in Atlanta, a tool used to conduct dollar store assessments in Baltimore, and a tool used to evaluate SNAP compliance among food retailers across multiple states [54–56, 63–67]. In addition, federal guidance and state-level requirements on the current WIC food packages informed the development of WIC readiness measures within the draft tool [53, 68].These prior adaptations informed the selection of food categories and WIC-aligned measures; however, none were adopted in full due to differences in retail format (e.g. dollar stores), product mix, and the need to align with updated WIC nutrition standards [54]. Drawing on variables from previously established food environment assessment tools, including food availability, variety, product placement and visibility, shelf-space allocation, marketing indicators, and WIC-eligible food categories, the draft of the NEMS-DS was developed iteratively through discussion meetings among the Dollar Store Research Work Group [53–56, 63–67]. Detailed descriptions of the tool’s development and validation will be reported in separate manuscripts.

The NEMS-DS tool captured several dollar store domains including: the exterior and the interior store conditions, food and beverage items, infant formula, child nutritionals, and a small number of non-food items (e.g., availability of cooking utensils and equipment, spices, cleaning products). The food categories included fruits and vegetables (fresh, canned, dried, frozen, juice), whole grains, dairy and dairy substitutes, meat and protein substitutes (e.g., eggs), infant food and formula, and ultra-processed foods [54–56, 63–67]. Depending on the food category, the tool assessed the availability, number of varieties, acceptability, placement (near the front entrance, at checkout, eye-level), shelf space measured in linear feet, and price of food items [54–56]. For each WIC category, the total number of food items that aligned with Federal WIC nutrition guidelines and state requirements was recorded [53, 68].

Content validity of the NEMS-DS was assessed through review by three external experts and pilot testing of the tool. Experts included community and research stakeholders working with dollar stores from Center for Nutrition & Health Impact, formerly the Gretchen Swanson Center for Nutrition, and federal agency partners from CDC. In addition, the draft tool was shared with the Dr. Glanz, developer of the original NEMS instrument. Experts evaluated the relevance and coverage of domains (e.g., availability, placement, pricing) and alignment with WIC food package requirements, study aims, and their feedback informed revisions. Following expert review, face validity and feasibility were assessed during pilot testing in six states (n = 30 stores, including 5 stores in the Lowcountry) from October 2023–March 2024, using structured written and verbal feedback from trained data collectors on clarity, comprehensibility, formatting, and item ordering.

Inter-rater reliability was assessed in a subset of pilot stores (n = 2 of 5) in the Lowcountry, South Carolina. Duplicate, independent audits were conducted on the same day by two trained data collectors. Agreement between raters was evaluated using percent agreement for categorical variables and intraclass correlation coefficients (ICCs) for continuous measures. Results indicated substantial to near-perfect agreement for categorical measures and good to excellent reliability for continuous measures, based on established benchmarks. Feedback from external reviewers and pilot data collectors was discussed during Dollar Store Research Work Group meetings to inform final revisions to the tool [54]. Upon incorporating all changes to the NEMS-DS tool, the final version was programmed into the Qualtrics survey platform to facilitate digital data collection when available for research sites. A trained data collector conducted NEMS-DS assessment in 13 sampled stores in the Lowcountry between July 2024 and March 2025, in parallel with data collection at other research sites by other data collectors.

To promote consistency across sites, data collectors completed a one-hour virtual training led by the study team, which included a detailed review of the standardized manual of procedures and in-store assessment protocols, along with guided examples of key measures. Data collectors were trained to follow standardized procedures, including moving systematically through the store, taking photographs, and returning to complete missing data. The training was recorded for later review, and data collectors were encouraged to reference the manual during data collection to support consistent application of the protocol (Appendix A). All data collectors also completed practice exercises using sample scoring exercises to apply the protocol and identify potential challenges prior to formal data collection. In addition, a pilot field assessment was conducted in a subset of stores to refine procedures and identify implementation issues; however, participation in pilot data collection was not required for all data collectors. Findings from pilot field assessments were reviewed by the study team, and calibration procedures were conducted by comparing scoring across assessors to identify inconsistencies. Clarifications and standardized decision rules were then provided to ensure consistent interpretation and application of key measures across sites. All data collectors were required to demonstrate competency in applying the standardized protocol prior to initiating data collection. Competency was assessed through completion of structured training exercises, including application of key measures. Data collectors were approved by the principal investigator of each site before proceeding to formal data collection. To maintain data quality throughout the study, ongoing quality assurance procedures were implemented through biweekly Dollar Store Research Work Group meetings, including periodic review of submitted data and team-based clarification of discrepancies as needed. This centralized approach ensured consistent application of the protocol across all sites, supporting inter-rater reliability as described below. The complete NEMS-DS instrument is provided in Appendix B. The University of South Carolina granted an exemption from the Institutional Review Board (IRB) for data collection in the Lowcountry.

## Measures

### Neighborhood and store characteristics

We collected neighborhood-level sociodemographic characteristics for each store’s census tract or block group. Variables included the racial/ethnic composition of the census tract, the rural/urban classification based on the 2020 U.S. Census, and block group ADI deciles from the 2022 state-level ADI dataset [59–62]. Store characteristics included chain brand, number of checkout counters, and whether SNAP benefits were accepted. Checkout count served as a proxy for customer exposure to point-of-sale marketing and store capacity [69]. Point-of-sale refers broadly to areas within a retail store including but not limited to checkout areas, where purchase decisions are influenced by factors, such as branded signage and product placements on endcaps or freestanding racks positioned near the front of the store to encourage impulse purchases [70, 71]. Financial transactions may or may not occur in point of sales. Checkout areas, by contrast, are the specific locations where payment is completed, defined as the counter and its immediate surrounding shelves [70, 71].

### WIC readiness

The WIC Readiness score assessed the alignment of store offerings with federal WIC food package standards across six categories: fruits and vegetables (fresh and frozen/shelf-stable), fruit juice, grains, dairy and dairy substitutes, proteins (e.g., ground meat) and infant foods and formula [53, 68]. For all non-infant items, a store received points based on the availability of different WIC-eligible varieties: 0 varieties = 0 points; 1–3 varieties = 1 point; 4–6 varieties = 2 points; 7–9 varieties = 3 points; 10–11 varieties = 4 points; 12 + varieties = 5 points. For infant items, 0 varieties = 0 points; 1–2 varieties = 3 points; 3 + varieties = 5 points. Sub-scores received an additional 2 points if at least 30% of store offerings in the category were WIC-eligible. Because subcategories contained differing numbers of items and potential varieties, the maximum potential sub-scores range from 16 points for juice to 53 points for produce. The overall WIC Readiness score ranges from 0 to 190 points. The score sheet is included in Appendix C.

### Healthy Food Availability (HFA)

In the original NEMS-S tool, the number of rows or shelf columns is used to count the variety of healthy food items available [55]. The variety assessment component of the HFA category of NEMS-DS was adapted primarily from the availability framework of the NEMS-S, rather than the NEMS-CS, since using the number of shelf rows or columns is better suited to the retail structure of dollar stores [55]. Although the NEMS-CS includes canned fruits and vegetables in its scoring matrix, it was designed for small, independently owned corner stores with limited aisle space and fewer product varieties [56]. In contrast, dollar stores more closely resemble limited-assortment grocery retailers in their use of standardized shelving, aisle organization, and high-volume packaged foods, making the NEMS-S framework for assessing variety based on shelf space and product rows more appropriate for capturing meaningful differences in healthy food availability [55]. Given differences in retail format, product mix and volume, and the need to align with updated WIC nutrition standards, none of the prior tools were adopted in full for developing NEMS-DS [54]. The NEMS-DS therefore retains the NEMS-S approach to assessing variety while expanding food categories to include shelf-stable and canned produce items relevant to dollar store offerings [55]. For subcategories within fruits, vegetables, and juice, 0 points were awarded if there were 0 varieties available; 1 point if 1–2 varieties were available, 2 points if 3–4 varieties were available; and 3 points if at least 5 varieties were available. For subcategories within grains, dairy, and proteins, the scoring depended on the particular item. Still, 0 points were generally awarded if 0 varieties were available, 1 point if at least 1 variety was offered, and some subcategories received an additional point if at least 2 varieties were available. The total HFA score ranged from 0 to 62 points (Appendix D).

### Marketing and visibility of healthy and unhealthy food items

Observers recorded whether selected healthy and unhealthy items (fresh or frozen produce, canned goods, salty snacks, sweet snacks, bottled water, and sugar-sweetened beverages) were displayed at checkout or visible from the store entrance. For each location, we also noted whether at least one healthy (fresh or frozen produce or canned goods) or at least one unhealthy item (salty or sweet snacks) was marketed or visible. Food categories included in the marketing and visibility assessment were selected based on the NEMS-S and NEMS-CS tools, the NEMS-DS pilot testing results, and some similar studies which identified items most commonly promoted at checkout and store entrances and most relevant to nutrition policy and consumer purchasing in dollar store settings (Appendix B) [55, 56, 65, 70, 72].

### Shelf space for healthy and unhealthy food items

Data collectors estimated the linear shelf space for healthy and unhealthy items in feet using a measurement application on their mobile devices: fresh, frozen, and canned fruits and vegetables and bottled water (classified as healthy items) and packaged salty (e.g. snack crackers, tortilla chips, potato chips, pretzels) and sweet snacks (e.g. candy bars, cookies, gummy candies, chocolate candies), sugar-sweetened beverages (e.g. soda, sports drinks, energy drinks, non-100% juice, sweetened coffee) (classified as unhealthy/highly processed items) [55, 56].

These categories were selected to distinguish nutrient-dense foods from calorie-dense, nutrient-poor products commonly emphasized in discount store environments consistent with prior nutrition retail audit frameworks, such as NEMS-S and NEMS-CS [55, 56, 65]. We calculated the total healthy shelf space, total unhealthy shelf space, healthy shelf space as a percentage of unhealthy shelf space, and water shelf space as a percentage of sugar-sweetened beverage shelf space. If healthy and unhealthy items received equal shelf space, these values would be 100%; if healthy items received less shelf space, these values would be lower than 100%. Lower percentages indicate stronger emphasis on calorie-dense, nutrient-poor foods (Appendix B).

### Statistical analysis

We summarized neighborhood and store-level characteristics using descriptive statistics. For all nutrition environment measures, including WIC readiness, HFA scores, marketing patterns, visibility, and shelf-space allocation we compared Lowcountry stores (n = 13) with stores from other research sites (n = 202). We conducted chi-squared tests for categorical variables and F tests for numeric variables. Analyses focused on identifying significant differences between rural Lowcountry stores and the broader sample, and on situating findings within a parallel study using NEMS-DS. This comparative approach enables interpretation of whether dollar stores in rural Lowcountry align with, exceed, or lag behind the national sample of dollar stores. All statistical analyses were conducted using R version 4.4.3 (R Foundation for Statistical Computing, Vienna, Austria).

## Results

Store-level and neighborhood characteristics are displayed in Table 1 for the 13 Lowcountry stores, compared with 202 stores across other research sites. Most Lowcountry stores were Dollar General (62%) and were located in rural, high-deprivation areas. All Lowcountry stores were classified as rural, compared with only 9% at other research sites (p < 0.001). This contextual difference is directly relevant to the study’s focus on nutrition environments and equity in rural areas. The statistically significant difference (p < 0.001) indicates that the observed contrast in rural classification between Lowcountry stores and stores at other research sites is unlikely to be due to chance supporting the interpretation that the Lowcountry sample represents a distinctly rural retail context rather than random variation across sites.

Almost 23% of sampled stores in the Lowcountry were Family Dollar, and 15% were Dollar Tree; stores in other research sites were more evenly spread across these three brands, but the differences were not statistically significant (Table 1). Five (38%) of the Lowcountry stores were located in low ADI neighborhoods, two (15%) in medium ADI neighborhoods, and six (46%) in high ADI neighborhoods; the majority (60%) of stores in other research sites were located in high ADI neighborhoods, although differences in ADI level by research site were not statistically significant (Table 1). Neighborhood racial compositions varied, with Lowcountry stores located in census tracts with higher percentages of Black residents and lower percentages of Hispanic residents; stores from other research sites were located in tracts that had significantly larger Hispanic populations (27% on average) (Table 1). All stores in the Lowcountry and 94% of stores in other research sites reported accepting SNAP benefits. Stores in the Lowcountry had significantly fewer checkouts than stores at other research sites (2.2 vs. 3.4, p < 0.001), indicating reduced capacity and greater concentration of point-of-sale marketing.

However, the distribution of sub-scores differed. Stores in the Lowcountry had substantially lower fruit and vegetable scores (7.3 vs. 13 out of a potential maximum of 53, p < 0.001), driven by limited frozen and shelf-stable produce offerings (4.3 vs. 12 out of a potential maximum of 39, p < 0.001). Although the Lowcountry dollar stores overall scored lower on total WIC readiness and had limited frozen shelf-stable produce, they actually had more fresh fruits and vegetables available than stores in other regions (3 vs. 0.92 out of a potential maximum of 14, p = 0.024). The remaining WIC sub-scores were generally low compared to the potential maximum for each sub-score and did not differ significantly between the Lowcountry and the other research sites. One notable exception is that stores in the Lowcountry had an average protein sub-score of 12, which is over 70% of the potential maximum of 17. Sub-scores for juice, grains, dairy, proteins, and infant items were uniformly low across regions, with no statistically significant differences (Table 2).

**Table 2:** WIC readiness scores by research site

Score and potential range	South Carolina (Lowcountry) (n = 13)		Other Research Sites (n = 202)		
	Mean (SD)		Mean (SD)		*p*-value
WIC Readiness Score (0–190)	51.0	(17.5)	51.6	(20.4)	0.92
Fruit and Veg. Sub Score (0–53)	7.3	(7.0)	12.6	(5.2)	< 0.001
Fresh Fruit and Veg. (0–14)	3.0	(4.8)	0.92	(3.1)	0.02
Frozen and Shelf-Stable Fruits and Vegetables (0–39)	4.3	(4.1)	11.6	(4.0)	< 0.001
Juice Sub Score (0–16)	6.2	(2.0)	6.12	(2.4)	0.88
Grains Sub Score (0–35)	5.0	(2.5)	4.98	(3.5)	0.98
Dairy Sub Score (0–29)	7.6	(2.0)	7.13	(3.3)	0.61
Protein Sub Score (0–17)	12.4	(1.8)	11.2	(3.1)	0.18
Infant Sub Score (0–40)	12.5	(9.4)	9.57	(10.0)	0.31

*p*-values are from one-way ANOVA (F-tests) comparing stores in South Carolina to stores in other research sites

Table 3 presents the average scores on the HFA score and its sub-scores. Stores in the Lowcountry had an average score of 23 (SD = 6.4) out of a potential maximum of 62, with no significant differences in total or sub-category scores compared with other research sites. As with the WIC readiness scores, Lowcountry stores scored well below the potential maximum on each of the HFA sub-scores comparing to the other research sites, except for the protein category, in which stores in the Lowcountry scored on average 11 (SD = 1.5) out of 15. Overall, HFA scores indicate constrained access to healthy food options, regardless of store location (Table 3).

**Table 3:** Healthy food availability scores by research site

Score and potential range	South Carolina (Lowcountry) (n = 13)		Other Research Sites (n = 202)		
	Mean (SD)		Mean (SD)		*p*-value
Healthy Food Availability Score (0–62)	22.9	(6.5)	25.6	(5.8)	0.11
Fruit Availability (0–6)	1.1	(1.4)	1.7	(1.1)	0.07
Veg Availability (0–6)	1.9	(2.0)	2.1	(1.0)	0.47
Juice Availability (0–12)	3.1	(1.3)	3.5	(1.5)	0.30
Grain Availability (0–10)	3.0	(1.2)	3.1	(1.6)	0.82
Dairy Availability (0–13)	2.7	(1.3)	3.2	(1.9)	0.39
Protein Availability (0–15)	11.2	(1.5)	12.0	(1.9)	0.11

*p*-values are from one-way ANOVA (F-tests) comparing stores in South Carolina to stores in other research sites

Table 4 shows that checkout marketing patterns strongly favored unhealthy foods. No Lowcountry stores displayed any healthy food items at checkout, and only four stores (31%) displayed bottled water. In contrast, all Lowcountry stores (100%) marketed unhealthy snacks and sugar-sweetened beverages at checkout, closely mirroring patterns in other research sites (Table 4). Sugary beverages were also prominently promoted through point-of-sale displays, including branded signage and product placements on endcaps and freestanding racks near store entrances.

**Table 4:** Marketing of healthy and unhealthy items at checkout by research site

	**South Carolina (Lowcountry)** **(n = 13)**		**Other Research Sites** **(n = 202)**		
	**N**	**(%)**	**N**	**(%)**	***p*-value**
At least one healthy item marketed at checkout	0	(0%)	7	(3.5%)	0.50
Fresh produce marketed at checkout	0	(0%)	7	(3.5%)	0.50
Frozen produce marketed at checkout	0	(0%)	1	(0.5%)	0.80
Canned goods marketed at checkout	0	(0%)	0	(0%)	
At least one unhealthy food marketed at checkout	13	(100%)	195	(97%)	0.50
Salty snacks marketed at checkout	12	(92%)	181	(90%)	0.76
Sweet snacks marketed at checkout	13	(100%)	195	(97%)	0.50
Water marketed at checkout	4	(31%)	111	(55%)	0.09
Sweetened beverages marketed at checkout	13	(100%)	189	(94%)	0.35

*p*-values are from chi-square tests comparing stores in South Carolina to stores in other research sites

Table 5 presents the frequency with which selected food and beverage items were visible from the front entrance. In the Lowcountry, only three stores (23%) had at least one healthy food and bottled water visible from the entrance, while 12 stores (92%) had at least one unhealthy food and a sugar-sweetened beverage visible from the entrance. Comparisons between Lowcountry stores and stores at other research sites showed no statistically significant differences in visibility patterns, underscoring consistent reinforcement of unhealthy options upon store entry (Table 5).

**Table 5:** Visibility of healthy and unhealthy items from front entrance by research site

	**South Carolina (Lowcountry)** **(n = 13)**		**Other Research Sites** **(n = 202)**		
	**N**	**(%)**	**N**	**(%)**	***p*-value**
At least one healthy food visible at entrance	3	(23%)	30	(15%)	0.43
Fresh produce visible at entrance	2	(15%)	23	(11%)	0.67
Frozen produce visible at entrance	2	(15%)	22	(11%)	0.62
Canned goods visible at entrance	1	(8%)	25	(12%)	0.62
At least one unhealthy food visible at entrance	12	(92%)	174	(86%)	0.53
Salty snacks visible at entrance	12	(92%)	159	(79%)	0.24
Sweet snacks visible at entrance	12	(92%)	172	(85%)	0.48
Water visible at entrance	3	(23%)	94	(47%)	0.10
Sweetened beverages visible at entrance	12	(92%)	150	(74%)	0.15

*p*-values are from chi-square tests comparing stores in South Carolina to stores in other research sites

The measurements of shelf space allocated to different food and beverage categories are displayed in Table 6. Shelf-space estimates reflected the total linear shelf length allocated to predefined product categories. In practice, products were often interspersed within the same shelves; in these cases, data collectors measured only the linear space corresponding to items within each target categories of healthier and less processed (e.g., fruits, vegetables, and water) versus less healthy and highly processed products (e.g., packaged salty and sweet snacks and sugar-sweetened beverages) and items outside these categories were excluded from measurement. On average, stores in the Lowcountry have 34 feet (SD = 27 feet) of shelf space stocking healthy food items, including fresh, frozen, and canned fruits and vegetables, while they have an average of 507 feet (SD = 379 feet) of shelf space stocking unhealthy packaged snacks. The average store’s healthy food shelf space is only 12% (SD = 13 percentage points) of that allocated to unhealthy food items. Shelf space for beverages is also heavily weighted toward unhealthy sugar sweetened beverages: stores have a mean of 29 feet (SD = 22 feet) of shelf space for water and 221 feet (SD = 174 feet) of shelf space for sugar sweetened beverages, and the average store devotes approximately one-fifth as much shelf space to water as it does to sugar-sweetened-beverages (s 21%, SD = 27 percentage points) (Table 6).

**Table 6:** Shelf space of healthy and unhealthy items by research site

	**South Carolina (Lowcountry)** **(n = 13)**		**Other Research Sites** **(n = 202)**		
	Mean (SD)		Mean (SD)		*p*-value
Total Healthy Food Shelf Space (feet)	34.3	(26.6)	35.7	(31.6)	0.88
Fresh Fruit	4.9	(8.5)	2.1	(7.7)	0.20
Fresh Vegetables	7.1	(12.2)	2.2	(8.2)	0.04
Frozen Fruit	1.7	(3.7)	2.0	(3.4)	0.78
Frozen Vegetables	5.1	(5.6)	4.8	(5.3)	0.83
Canned Fruit	3.9	(4.4)	10.3	(10.8)	0.03
Canned Vegetables	11.6	(6.6)	14.3	(14.2)	0.49
Total Unhealthy Food Shelf Space (feet)	507	(379)	352	(296)	0.07
Salty Snacks	251	(192)	156	(140)	0.02
Sweet Snacks	256	(188)	196	(168)	0.22
Healthy Food Shelf Space as Percent of Unhealthy Food Shelf Space	12.0	(13.1)	22.2	(46.3)	0.43
Water Shelf Space (feet)	29.2	(22.3)	35.2	(32.2)	0.51
Sweetened Beverages Shelf Space (feet)	221	(174)	161	(174)	0.23
Healthy Beverage Shelf Space as Percent Unhealthy Beverage Shelf Space	21.5	(27.4)	36.5	(50.4)	0.29

*p*-values are from one-way ANOVA (F-tests) comparing stores in South Carolina to stores in other research sites

## Discussion

This study provides one of the first comprehensive assessments of the nutrition environments and WIC readiness of dollar stores in rural and high-deprivation communities of the Lowcountry, SC, using the newly developed NEMS-DS tool. By comparing 13 rural dollar stores in the Lowcountry, SC with 202 dollar stores across the other research sites (New York, Texas, Illinois, Florida, Maryland, Georgia, Louisiana, Michigan, Southern California), this study highlights structural barriers to healthy food access within a rapidly expanding retail sector that plays an increasingly central role in community nutrition access in low-income rural communities [34, 39, 44, 73–78].The higher proportion of rural stores located in the Lowcountry sample, compared with other research sites, also is meaningful, as rural retail environments are systematically associated with greater food access constraints, fewer retail options, and higher exposure to less-healthful products compared with urban settings [73, 74].

Across all domains (availability, visibility, marketing, and shelf-space allocation) Lowcountry dollar stores reflected a food environment with limited availability of nutritious foods and dominant placement of ultra-processed products, consistent with prior research [36–38, 46, 79]. Although there was slightly higher availability of fresh produce in some Lowcountry stores, likely due to Dollar General’s fresh initiatives, these offerings remained insufficient to counter less-healthful options [80]. Overall, healthy items accounted for only a small fraction of total shelf space, and unhealthy snacks and sugar-sweetened beverages dominated checkout and entrances displays, patterns known to influence impulse purchasing and dietary behaviors [36–38, 40, 42, 46, 79].

The smaller number of checkout lanes observed in Lowcountry stores may further intensify this exposure, as promotional materials are concentrated within fewer transaction areas, increasing the density and visibility of point-of-sale marketing per checkout. This spatial concentration may amplify customer exposure to marketed products even with fewer checkout locations [70, 71, 77]. These findings highlight consistent national trends in the prominence of ultra-processed products at points of sale in dollar stores [70, 71, 77].

Importantly, shelf-space allocation in retail settings is often shaped by manufacturer–retailer agreements, whereby companies pay for premium placement in high-visibility locations, endcaps, and high-traffic locations, which may contribute to the disproportionate prominence of ultra-processed foods observed in sampled stores in this study [70, 71, 81–83]. These industry practices favor larger companies with greater promotional resources, while healthier products may receive less visibility due to the limited capacity of health-oriented manufacturers to compete for paid placement [81–83]. Therefore, these industry-level arrangements may limit store managers’ autonomy in adjusting product mix toward healthier offerings, even when there is community demand for improved food access [81–83]. This underscores the need for policy and programmatic approaches, such as WIC authorization, procurement partnerships, and financial incentives to support healthier food stocking in small and nontraditional retail settings.

WIC readiness scores were low in both the Lowcountry and comparison research sites, indicating that many key WIC food groups, such as fruits, vegetables, and whole grains, remain scarce. Although protein items were more available, overall readiness profiles suggest that substantial improvements are needed for dollar stores to meet WIC standards. While dollar stores accept SNAP, many do not qualify as WIC-authorized vendors; consequently, low WIC readiness represents a missed opportunity to improve access to nutrient-dense foods for low-income rural families with young children [84–86]. The low rate of WIC participation in SC (1.9% in 2024) could also reflect limited access to WIC-authorized stores for redeeming this benefit [87].

Findings from this study are consistent with prior research using tools to assess WIC related food availability in other retail settings [88–90]. Studies such as the Texas Nutrition Measures Survey–WIC has documented limited availability of WIC-approved foods, particularly fruits, vegetables, and whole grains, among small and nontraditional retailers serving low-income populations [88–90]. However, the NEMS-DS further documents the disproportionate placement of ultra-processed foods in high-visibility locations, offering additional insight into structural factors shaping food choice in dollar store environments. While prior tools, were designed primarily to measure availability and price of WIC-eligible foods in grocery and small-store formats, the NEMS-DS extends this work by capturing marketing, visibility, and shelf-space allocation in dollar stores.

The structural barriers observed in Lowcountry dollar stores have essential implications for nutrition equity. From a practice standpoint, the NEMS-DS is intended to be administered by any individual that seeks to assess the food environment, including researchers, students, community leaders, and public health practitioners. Data generated from NEMS-DS can inform policymakers by identifying inequities in dollar stores food access (e.g., limited healthy food availability and marketing of unhealthy items at checkouts), and guiding policy decisions related to nutrition assistance programs, such as WIC vendors expansion include dollar stores, retailer incentives that support stocking of healthier and WIC-authorized items, and community food interventions with sustainable outcomes [91, 92]. Procurement partnership with local distributors or farmers could also increase access to nutritious foods by expanding the availability of fresh and culturally relevant produce in rural communities [93–95]. Some studies also show effective results employing similar strategies and interventions in increasing the availability and access to nutritious products in small stores [91, 92]. These multilevel strategies are consistent with the Community Nutrition Environment and Social-Ecological Models, which emphasize the role of structural and environmental determinants in shaping dietary behavior [57, 58].

This study has several strengths, including a multi-site comparative design, and detailed measures of shelf space, marketing, and visibility. These metrics go beyond typical food environment assessments and provide high-resolution insight into rural nutritional landscapes. However, several limitations should be acknowledged. Because this study employed a cross-sectional design, findings cannot establish causal relationships between store environments and health outcomes. The Lowcountry sample also was relatively small and entirely rural; although it represents most operational rural dollar stores in the region, the limited sample size restricts the ability to examine variation across counties or store types. Additionally, unequal sample sizes across sites may have influenced comparisons and limited statistical precision. Because the comparison sample included predominantly urban and suburban stores and sample sizes varied across sites, observed differences may partially reflect contextual variation rather than regional effects alone. Preliminary inter-rater reliability was assessed only in a subset of pilot stores in the Lowcountry and was not evaluated across all research sites, due to funding and time constraints, which may limit generalizability. Additionally, because reliability testing was conducted in rural retail settings, findings are most directly applicable to similar rural contexts. Future studies should assess reliability across urban and suburban retail environments to further assess generalizability.

As NEMS-DS is a newly developed instrument, continued validation across diverse geographic and retail contexts is warranted, and findings should be interpreted within the context of ongoing instrument development. The NEMS-DS tool also is relatively narrow in its measurement of the marketing, visibility, and shelf space of healthy and unhealthy food items, focusing only on produce and packaged snacks. But many other items could be considered healthy (e.g., proteins and dairy) or unhealthy (e.g., sugar-sweetened cereals). Additionally, this study does not capture transportation barriers, proximity to dollar stores, active community food programs, regional economic conditions, household food preparation capacity, or consumer purchasing behavior, all of which are important determinants of diet quality [26, 96–100]. While data collection was completed in the majority of stores, there were a small number of instances where assessments could not be fully completed due to time constraints, interruptions from store personnel, or requests for data collectors to leave. We did not observe consistent systematic patterns in missingness by store characteristics or location. However, anecdotal reports from data collectors suggest that smaller, more crowded stores with limited space and higher customer traffic posed greater practical challenges, particularly for measures collected later in the assessment. Finally, as with all field-based data collection, while this measure is subject to some practical constraints, we believe the procedures used supported its reasonable reliability in practice. In particular, shelf-space measures relied on a mobile application and required data collectors to estimate linear space for predefined product categories within shared shelving areas. Although formal reliability testing for shelf-space measures was conducted only in a limited subset of pilot stores only in SC, several additional procedures were implemented to enhance consistency, including standardized training, validation of the mobile measurement application against a ruler, and photographic documentation for verification. In practice, products were often interspersed within the same shelves, requiring data collectors to estimate only the portion of space occupied by relevant items. While this may have introduced some measurement error and contributed to missingness, these challenges were not systematic across store types. As such, shelf-space measures provided a meaningful indicator of the relative allocation of retail space to healthier and less processed versus less healthy and highly processed product categories and were sufficient to capture variation in the in-store food environment. The categories were intentionally defined to be relatively broad and data collectors were trained on inclusion and exclusion criteria for each measure. Data collectors instructed to include all subtypes within each category (e.g., all canned fruit) which helped to simplify classification decisions in the field. However, we acknowledge that applying these procedures in settings where products are highly intermixed, may have posed practical challenges.

The present findings advance understanding of how the food environments of dollar stores in Lowcountry, SC, may influence access to nutritious foods among rural communities. Future work could also explore whether corporate commitment to healthier stocking could be translated into sustainable improvements in access to nutritious foods and tracking progress toward becoming a WIC vendor, particularly in rural regions. Finally, integrating NEMS-DS data with sales and qualitative inquiry on consumer behavior could provide a more complete picture of how dollar stores’ food environments could impact diet-related health outcomes in rural settings.

## Conclusion

This study provides one of the first comprehensive assessments of rural dollar store food environments and WIC readiness in the Lowcountry of SC using the NEMS-DS tool. Findings reveal limited availability of nutritious foods, low alignment with WIC standards, and disproportionate promotion of ultra-processed snacks and sugar-sweetened beverages, reflecting broader structural inequities in high-poverty rural communities. These findings contribute to the growing literature on rural food environments and demonstrates the value of the NEMS-DS for evaluating healthy food access in nontraditional store settings. Multi-level strategies, such as expansion of WIC vendor authorization, nutritious product placement standards, and strengthened community partnerships may help improve the nutritional landscape of these stores. Enhancing the availability of healthy food in dollar stores offers a promising opportunity to advance food justice and reduce diet-related health disparities in rural areas.

## Supplementary Information

Supplementary Material 1.

## Data Availability

All data supporting the findings of this study are included within this manuscript. Data from comparison research sites are available upon reasonable request to the corresponding author and with approval from Dr. Rachael Dombrowski, the principal investigator of the main multi-state study.

## Abbreviations

ADI: Area Deprivation Index
ANOVA: Analysis of Variance
CDC: Centers for Disease Control and Prevention
CNE: Community Nutrition Environment
HER: Healthy Eating Research
HFA: Health Food Availability
HFR: Healthy Food Retail
ICC: Intraclass correlation coefficients
IRB: Institutional Review Board
MBAT: Market Basket Assessment Tool
NEMS-CS: Nutrition Environment Measure Survey-Corner Stores
NEMS-DS: Nutrition Environment Measure Survey-Dollar Stores
NEMS-S: Nutrition Environment Measure Survey-Stores
NOPREN: Nutrition and Obesity Research and Evaluation Network
RWJF: Robert Wood Johnson Foundation
SC: South Carolina
SD: Standard Deviation
SEM: Social Ecological Model
SNAP: Supplemental Nutrition Assistance Program
U.S: United States
WIC: Women, Infant, Children program
