# Supplementary material for: A nutrition environment measures survey for dollar stores (nems-ds) in rural south carolina: assessing nutrition equity and WIC readiness
Source: BMC Public Health. 2026 Apr 29;26:1803. doi: 10.1186/s12889-026-27446-6 (PMC13235116; doi:10.1186/s12889-026-27446-6)
Supplement: Supplementary file 1 — Supplementary Material 1. [file 12889_2026_27446_MOESM1_ESM.docx]

Appendix A

**Nutrition Environment Measures Survey for Dollar Stores**

**(NEMS-DS)**

**Data Collection Protocol and Manual**

**November 2025**

[**Table**](https://www.fns.usda.gov/wic/wic-food-packages-regulatory-requirements-wic-eligible-foods) **of Contents**

[Background 1](#_Toc164939562)

[Objective 3](#_Toc164939563)

[Data Collection Protocol & Guidance 4](#_Toc164939564)

[General: 4](#_Toc164939565)

[Time of assessment & Number of store visits 4](#_Toc164939566)

[Preparation 4](#_Toc164939567)

[While in the store: 4](#_Toc164939568)

[After visiting a store: 4](#_Toc164939569)

[Store Layout and Appearance 5](#_Toc164939570)

[Store Layout 5](#_Toc164939571)

[Store Type 5](#_Toc164939572)

[Exterior 5](#_Toc164939573)

[Trash/Debris 5](#_Toc164939574)

[Graffiti 6](#_Toc164939575)

[Loitering 6](#_Toc164939576)

[Panhandling 6](#_Toc164939577)

[Nearby bus stop 6](#_Toc164939578)

[Shopping cart rails 6](#_Toc164939579)

[Landscaping 6](#_Toc164939580)

[Security 6](#_Toc164939581)

[Interior 6](#_Toc164939582)

[Cleanliness 6](#_Toc164939583)

[Security 7](#_Toc164939584)

[Number of Checkouts 7](#_Toc164939585)

[Promoted Items 7](#_Toc164939586)

[Checkout Items 7](#_Toc164939587)

[Food Environment Assessment 7](#_Toc164939588)

[Overview & Definitions 7](#_Toc164939589)

[Amount 8](#_Toc164939590)

[Price 8](#_Toc164939591)

[Size 8](#_Toc164939592)

[Number of Varieties 8](#_Toc164939593)

[Location 8](#_Toc164939594)

[Produce and Juice 9](#_Toc164939595)

[Allowable Fruits and Vegetables (Fresh, packaged (dried/canned), or frozen) 9](#_Toc164939596)

[Not Allowed 10](#_Toc164939597)

[Juice Nutrition Requirements 10](#_Toc164939598)

[Protein and Protein Substitutes 10](#_Toc164939599)

[Allowable Fish (Canned Only) 11](#_Toc164939600)

[Canned Fish Nutrition Requirements 12](#_Toc164939601)

[Allowable Nut and Seed Butters 12](#_Toc164939602)

[Allowable Mature Legumes (Dry Beans and Peas) 12](#_Toc164939603)

[Allowable Eggs 12](#_Toc164939604)

[Dairy and Dairy Substitutes 12](#_Toc164939605)

[Allowable Types of Cow’s Milk 13](#_Toc164939606)

[Allowable Cheese 14](#_Toc164939607)

[Cheese Nutrition Requirements 14](#_Toc164939608)

[Yogurt Nutrition Requirements 14](#_Toc164939609)

[Whole Grains 15](#_Toc164939610)

[Allowable Whole Wheat Bread/Whole Grain Bread/Other Whole Grains 16](#_Toc164939611)

[Whole Grain Nutrition Requirements 16](#_Toc164939612)

[Allowable Breakfast Cereal 17](#_Toc164939613)

[Breakfast Cereal Nutrition Requirements 17](#_Toc164939614)

[Ultra-Processed Foods 17](#_Toc164939615)

[Infant Formula and Food 18](#_Toc164939616)

[Allowable Infant Formula 19](#_Toc164939617)

[Infant Formula Nutrition Requirements 19](#_Toc164939618)

[Allowable Exempt Infant Formula (Hypoallergenic/Specialized/Premature) 19](#_Toc164939619)

[Exempt Infant Formula Nutrition Requirements 19](#_Toc164939620)

[Allowable Nutritionals 19](#_Toc164939621)

[Nutritionals Nutrition Requirements 20](#_Toc164939622)

[Allowable Infant Cereal 20](#_Toc164939623)

[Infant Cereal Nutrition Requirements 20](#_Toc164939624)

[Allowable Infant Food - Fruits and Vegetables 21](#_Toc164939625)

[Allowable Infant Food - Meat 21](#_Toc164939626)

[Measuring Shelf Space 21](#_Toc164939627)

[Non-Food Items 22](#_Toc164939628)

#

# Background

The spread of dollar stores in the United States (U.S.), particularly within impoverished communities and communities of color, has raised concerns among residents, policy makers, and public health professionals about their potential impact on community health, economic development, and food security. Recent assessments of dollar stores in Michigan, Illinois and Maryland have found a limited selection of food items, and many highly processed and packaged foods. On the other hand, new models such as “DG Fresh” (which is a Dollar General store with fresh and frozen produce, fresh meat, dairy, and grain products) show promise in shifting the landscape of many of these stores (Troy, 2021).

A lack of healthy food options in communities can exacerbate food insecurity and contribute to poor health outcomes, particularly in low-income communities where dollar stores are disproportionately present and full-service grocers are not readily available (National Institute on Minority Health and Health Disparities, 2023; Mitchell, Smith and Holmberg, 2023). Furthermore, many low-income families who participate in federal nutrition support programs such as the Special Supplemental Nutrition Program for Women, Infants, and Children (WIC) and the Supplemental Nutrition Assistance Program (SNAP) may turn to dollar stores to redeem their benefits if there are no supermarkets or grocery stores present in their communities. While most dollar stores accept SNAP, few dollar stores are WIC authorized (Wallace et al., 2020; Hudak et al., 2020), leaving limited options for families to redeem their WIC benefits in many urban and rural communities.

In response to the rapid expansion of dollar stores, nearly 30 municipalities have implemented policies that restrict where they can locate and specify what they should sell (e.g., must dedicate a certain proportion of shelf-space to specific types of healthy foods) (McCarthy et al., 2022). To date, research which examines the internal dollar store food environment is both limited (Caspi et al., 2021; Wallace et al., 2020; Hudak et al., 2020; Racine et al., 2016), and idiosyncratic, making comparisons between settings difficult. Given the dramatic growth of dollar stores throughout the country, an assessment tool and scoring systems that permit direct and systematic comparisons between dollar stores in different settings, and that would allow for an assessment of key features, such as readiness for participation in federal nutrition assistance programs (e.g., WIC), is both crucial and timely.

Accepting WIC benefits in dollar stores is a potential strategy that could benefit millions of Americans. Since 1972, WIC has helped to ensure the provision of nutrition education, supplemental foods and health care referrals for low-income women, infants, and children (U.S. Department of Agriculture, 2023a). From 2016 to 2019, the total number of *WIC participants* as a proportion of WIC *eligible* participants declined from 57% to 50% (Gray et al., 2022). Recent efforts at the federal and state levels have attempted to eliminate this gap through innovative strategies of WIC enrollments and through modernization of WIC services, such as improving the retail shopping experience (U.S. Department of Agriculture, 2023b). These efforts within grocery retailers include more easily identifying WIC products within grocers, supporting easier checkouts, providing online shopping opportunities, and providing training for store staff to assist WIC consumers (U.S. Department of Agriculture, 2023b; Federal Register, 2022). While these modernization efforts are commendable, more strategies may be needed to increase and sustain WIC participation among families most in need. These strategies could include the enrollment of additional WIC vendors, such as dollar stores, which are located in many low-income communities throughout the country (Mitchell, Smith and Holmberg, 2023).

Improving the quality of research within dollar stores is essential to improving access to healthier foods, especially for those eligible for and participating in nutrition assistance programs. A systematic tool and scoring system for assessing the characteristics of dollar stores will assist researchers, community members and policy makers in creating more appropriate policies and programs that align with community needs and could promote an alliance with dollar stores to increase healthy food access and improve nutrition security. In addition to increasing our understanding of the dollar store food environment in the present moment, developing and employing a dollar store specific tool and scoring system that aligns with the new WIC guidelines will determine some aspects of readiness of dollar stores to accept WIC (Gittelsohn et al., 2012).

Structured observation instruments exist for assessing the retail food environment. The current nutrition environment measures survey for stores (NEMS-S) (Glanz et al., 2007) and corner stores (NEMS-CS) (Cavanaugh et al., 2013) have been effective in measuring food availability, quality and price in supermarkets, other large format grocers, and small food stores. These structured instruments are centered around data collection by a trained observer. However, the NEMS-S and NEMS-CS produce incomplete or inaccurate reporting for the dollar store retail environment, given the experience of several members of the research team (Dombrowski, Bode, Gittelsohn, Sundermeir and Kaur), as mentioned above. Furthermore, a recent systematic review found no adaptations of the NEMS-S or NEMS-CS for use in dollar stores (Glanz et al., 2023). Previous assessments in dollar stores using the NEMS-S conducted in Michigan (2022) and Illinois (2023) revealed an inability to effectively assess quality and price of food - given a heavier reliance on shelf stable and frozen foods in most dollar stores throughout the country. Additionally, the NEMS-S and NEMS-CS do not currently account for non-food items and marketing and availability of ultra-processed foods within dollar stores (e.g., product placement and signage).

This project seeks to address these gaps by developing a modified survey tool that determines how and in what ways dollar stores align with the federal WIC food package - by assessing availability, affordability and quality of those key WIC food items. Additionally, the tool will include objective measures of the dollar store nutrition environment, such as the availability and prices of healthy food options, the wide array of ultra-processed food items available, and the presence of food marketing. **This study will focus on testing the feasibility and effectiveness of the modified structured observation tool (NEMS-DS) in 200 dollar stores across 10 states and will also develop a scoring system to determine WIC readiness and other healthy retail metrics for ease in determining the landscape of the dollar store food environment.**

# Objective

The goals of the proposed project are to develop and pilot an observational tool within dollar stores that can be utilized in 10 sites (N=200 stores) to determine readiness for dollar store participation in WIC and other healthy retail metrics. This study will test hypotheses of dollar store readiness to participate in WIC and other healthy retail metrics through a) accurately assessing the in-store food environments of dollar stores, b) assessing food availability, affordability, and access across the 10 sites and c) aligning those measures to WIC readiness, healthy food availability, community resourcefulness and other healthy retail metrics via a scoring mechanism. We will also test whether community sociodemographic characteristics (e.g., racial/ethnic makeup, income levels) across each site predict scores within stores. The specific aims of this project are to:

1. Collaboratively develop a new dollar store food environment assessment tool (NEMS-DS) and pilot the instrument in six diverse geographic locations to improve the accuracy and face validity of the instrument.
2. Utilize the revised NEMS-DS to conduct dollar store food environment assessments at 10 diverse sites (N=200 dollar stores) across the United States to determine capacity for becoming certified by the Special Supplemental Nutrition Program for Women, Infants, and Children (WIC) and by examining whether minimum federal food package requirements are met.
3. Create a scoring system that adequately illustrates the internal dollar store food environment and readiness for participating in WIC, as well as stores’ adherence to current SNAP requirements and other food access metrics (e.g., ultra processed foods, non-food items, etc.) in comparison to population sociodemographic characteristics.

# Data Collection Protocol & Guidance

## General:

Data collectors should go in pairs to collect data – do not complete store visits alone for any reason. The tool is generally organized in the order that it should be completed for ease of navigating the store. Shelf space measurement should be done towards the end of data collection. Once data collectors are fully familiar with the tool, it should take approximately 30-45 minutes to complete the assessment.

### Time of assessment & Number of store visits

Complete store assessments between 10 am and 4 pm. This helps to ensure that items have been stocked for the day and are not sold out. In small stores, it also helps to ensure that you are not in the way during a busy time. Most stores will be open during these hours. Occasionally, stores may be temporarily closed (e.g., if the only store staff on site had to close the store for an emergency). For stores that appear open for business but temporarily closed at the time of the visit, a second visit to the site will be made.

### Preparation

- Before you go to the store, fill in the following at the top of each page: data collector names, store address, date, and time for each store you plan to visit
- Plan your route (which stores will you visit in which order)
- Ensure you have all materials that you need, including list of store addresses to visit, # of assessment forms plus 3 extras, this protocol, clipboard, pencils.
- Bring water and your phone
- Notify a roommate, friend, partner, or other close contact of what you are doing and how long you will be gone. Tell them to contact your supervisor if you do not return within an hour of the time you specified and cannot be reached by phone.

### While in the store:

- When collecting data with a partner: Use different methods to split up the survey between you and your partner. You may divide up the survey so that one surveyor looks for items in one section and the other surveyor looks for items in another section.
- Use techniques to keep your time in the store brief and efficient. Learn which types of foods are likely to be near each other. Make pencil marks and notes for items that then can be confirmed and finalized once outside the store.
- If store staff ask you to leave for any reason, please do so immediately. Document that you were asked to leave and consider when you might be able to return in the future on a different day, time to make another attempt.

### After visiting a store:

Once you walk out of the store: Check your work. Finalize responses on the assessment tool. Ensure all items on the form are completed and legible (if using paper).

If you are completing a paper form, you are responsible for entering the data into the Qualtrics platform. Please take this step immediately.

# Store Layout and Appearance

*(Tool pages 3-4)*

## Store Layout

Look for cues as to whether the store accepts food stamps, also called the Supplemental Nutrition

Assistance Program (SNAP) or EBT (Electronic Benefits Transfer). This information may be

posted on signage at the entrance of the store or at the checkout register. If information is not

readily available, ask the store clerk whether SNAP/EBT (food stamps) are accepted.

Look for cues as to whether the store accepts WIC. These are provided to participants of the Special Supplemental Program for Women, Infants, and Children (WIC) for the purchase of specific food and beverage items. This information may be posted on signage at the entrance of the store, at the checkout register, or on shelf tags near WIC-approved items (e.g., some cereals, milk, cheese, eggs). If information is not readily available, ask the store clerk whether WIC is accepted.

## Store Type

Data collection will take place in the three major dollar store brands: Family Dollar, Dollar Tree, and Dollar General. Other discount stores, chain and locally owned, will not be included. Select which store type you are assessing

## Exterior

Does the store have parking on-site? On-site parking can include a lot, or a parking structure/deck associated with the store. It does not matter if the parking is “paid”, “free”, “valet” or “self-park” this should be included. It does not matter if parking is shared with other neighboring businesses such as in a strip mall this should be included. Designated lines marking spaces do not have to be present to be considered on-site parking. Street parking (free or metered or designated for the store specifically) should not be recorded as on-site parking, as it is not directly associated with the store. If parking for the store is present in a lot or parking structure located a few doors down (i.e., not adjacent to) from the store and is clearly designated for store patrons it should be included, even if this parking lot is shared with other businesses. If the store has on-site parking, circle “1” for yes. If not, circle “0” for no.

### Trash/Debris

Trash/debris includes paper, packaging, bottles, cans, broken glass, cigarette packs and other items of refuse found (excluding cigarette butts) at the facility entrance. The facility entrance area includes the store's entrance and the sidewalk immediately in front of it. If the store has ‘little to no’ garbage, circle “0” for no. If the store has a moderate or high amount of garbage that affects the feeling of cleanliness or safety of the store, circle “1” for yes.

### Graffiti

Graffiti, tagging, or “unapproved” writing, includes painted or drawn signs or symbols (e.g., gang insignia) on the building and/or exterior property visible from the street or parking lots. Do not include painted murals or public art. If the store has ‘little to no’ graffiti, circle “0” for no. If the store has a moderate or high amount of graffiti that affects the feeling of cleanliness or safety of the store, circle “1” for yes.

### Loitering

Loitering is standing or waiting around [idly](https://www.google.com/search?sca_esv=570532409&rlz=1C5CHFA_enUS882US883&q=idly&si=ALGXSlYftE7XViDpYdZ4PMnebYlv_v8DnMlualVmTw2sAzT0rrWu8bV-WFOH0e0I8drJ8xjFTzT_cqUhW5NkkN7qeqXljQLa4g%3D%3D&expnd=1) or without apparent purpose; circle “1” for yes.

### Panhandling

Panhandling is asking/begging for money; circle “1” for yes.

### Nearby bus stop

From the entrance and/or parking lot of the store, can you see a bus stop? There might be a sign or a shelter indicating that it is a bus stop. Circle “1” for yes.

### Shopping cart rails

Shopping cart rails store shopping carts. They may be in the parking lot or right outside the front entrance. Be sure the shopping cart rails don’t belong to another store nearby (like a supermarket). Circle “1” for yes.

### Landscaping

Note any landscaping outside of the store, either along the building or in the parking lot. This includes shrubs/bushes, trees, and flowers. Circle “1” for yes.

### Security

Look for cameras attached to the building facing the store entrance and/or parking lot.

Look for security bars: bars include those permanently covering the outside of the store windows. Do not include the folding metal bars/cages that can be temporarily pushed aside during hours of business. Circle “1” for yes if security items are present.

## Interior

### Cleanliness

Store cleanliness includes trash/debris inside the store, cluttered floors (e.g., boxes stacked up, inventory sitting in the aisles, blocked aisles, recycling/trash stacked up), a foul odor, and panhandling. Circle “1” for yes.

### Security

This refers to whether a store has security mirrors, cameras, guards, and/or monitors inside. Many times, these mirrors can be found on the ceiling or in other high places of the store. If security cameras, mirrors, or monitors are not visibly obvious, data collectors should not ask a store employee if security measures are taken. Security guards/personnel usually stand next to the front door and are typically wearing official security attire. Staff members/employee stationed near the door (in the same manner as a security guard) do not count. If the data collector can see the security installations (e.g., mirror, guard, camera), circle “1” for yes. If not, circle “0” for no. You will also indicate whether the shelving is so high that you can’t see across the store. This is important because if shelving is high, staff cannot see across the store/monitor customers etc. Circle “1” for yes.

### Shopping cart rails

You will also look for shopping cart rails inside the store. They are usually located just inside the front entrance to the store. Circle “1” for yes.

### Number of Checkouts

Intentionally walk by the checkout area to ensure a true count of each type of checkout. A checkout lane is defined as an intentional space where customers can pay for their goods. The checkout may be self-checkout style where the customer interacts with a computer to pay for their goods. The checkout may be a traditional checkout where a store employee facilitates the way a customer pays for their goods face to face. An overflow checkout may look like a designated customer service desk where a store employee serves as a designated window or counter where people go to resolve a customer concern and where people might go to checkout, but it is not the traditional checkout lane or a self-checkout lane. Count all checkouts, regardless if they are staffed or not.

### Promoted Items

This section is asking about items you can see from the front entrance of the store, right when you walk in. Check “yes” if the item is visible, and “no” if it is not.

### Checkout Items

This section asks about items for sale at the checkout counter. This includes things displayed at the cash register (next to, below) and on the endcaps as you enter a checkout aisle. Check “yes” if the item is present, and “no” if it is not.

# Food Environment Assessment

### Overview & Definitions

Once you have completed the Cover Page and General Information section of the form you can move on to the food environment assessment components. The assessment is divided into six sections: produce and juice, proteins/meats/protein substitutes, dairy, whole grains, ultra—processed foods, and infant food and formula. There is a seventh, final section for non-food items.

According to the USDA, perishable foods are either frozen or fresh, un-refrigerated or refrigerated, food items that will spoil or suffer significant deterioration in quality within 2 to 3 weeks. Non-Perishable foods are canned, dried, or shelf-stable items that will NOT spoil or suffer significant deterioration in quality within 2 to 3 weeks. The remaining sections of this training manual describe in detail what would count as variety in each of the staple food categories. Many of the food items captured by this tool are allowable foods in the federal WIC program and therefore need to meet certain nutritional requirements. Data collectors will identify and record information on foods and/or beverage varieties that would count toward the new federal WIC standards through reviewing food items and cross-referencing nutrition facts labels and ingredient lists with the specific nutrient thresholds and/or ingredient stipulations outlined below.

### Amount

Amount refers to the depth of stock for each item. You will circle there is 1 item in stock, 2 items in stock, or 3+ (3 or more) of the item in stock. If an item is unavailable, meaning that it is not present in the store AND there are no signage/shelf talkers indicating that there is a location for it, leave that line blank. If there is a clear location for an item (e.g., shelf talker/price tag) that is in the instrument, but it appears to be sold out circle “0” under amount.

### Price

Record the prices for the items indicated – note, we are not collecting prices for every food item. Items that we are not collecting price for either do not appear on the table or are grayed out**. For the items indicated, record the largest size with the lowest price**. Do not include sale prices.

### Size

Record the size of the item and how it appears on the package (oz, lbs, etc.). **Again, you are recording the largest size with the lowest price (not including sales**), unless otherwise indicated in specific sections. Document single-serve items if they are the only size available and otherwise meet the categorical criteria. Document multi-pack products by the individual size and count such as 4 x 4 oz.

### Number of Varieties

At the end of several of the sections, there will be fields to complete for the number of WIC-approved and the total number of varieties overall. This will allow us to calculate the proportion of WIC-approved varieties in each food category. If WIC -approved varieties are present, this should be a simple addition of the number of rows completed for a certain category (e.g., frozen vegetable varieties). For total varieties, conduct a simple count of the total number of varieties available (included WIC-approved). Variety is irrespective of the brand. For example, if there are 2+ brands of canned peaches that are non-WIC approved due to containing sugar, you would count “1” for the variety of peaches despite there being more than one brand.

### Location

Where noted, document the placement of specific food items or categories by circling either “EL” for eye-level, “H” for high, “SD” for special display, “L” for low, or write in another location under “Other.” Eye-level means that the item is right in your view, and you do not need to bend down/reach up high to get it. High refers to items you must *reach to get*. A special display may be a separate shelf or table set-up to feature a certain item. For example, during Halloween there might be a special display of Halloween candy, separate from the rest of the candy in the store, in a prominent location (e.g., near the front entrance). Low refers to items you must *bend down to get*.

## Produce and Juice

*(Tool pages 5-12)*

For fruits and vegetables, data collectors will document the *availability* of each type of fruit or vegetable in all its forms (fresh, frozen, canned, dried, 100% juice, and juice concentrate) if it meets the nutritional requirements for its respective form. Each row includes blank spaces to fill in a fruit or vegetable and complete the row for that specific fruit or vegetable in different forms. Data collectors should refer to the below nutritional requirements when deciding whether to count a food item. If a juice variety is available in both perishable and shelf-stable form, document both and note which form in the “Fruit/Vegetable Type” field. For example, if there was refrigerated orange juice and shelf-stable orange juice, you would make one entry for “Refrigerated orange” and a second for “Shelf-stable orange” and select “Refrigerated or Shelf-Stable 100% Juice” for the Form.

### Allowable Fruits and Vegetables (Fresh, packaged (dried/canned), or frozen)

**Fresh Fruits and Vegetables**

- Any variety of fresh whole or cut fruit without added sugars. Please note acceptability of fresh food items by circling “1” if more than 50% of fresh produce items is not spoiled, high quality (e.g., no bruises, mold, etc.).
- Any variety of fresh whole or cut vegetables without added salt, fats, or oils. Please note acceptability of fresh food items by circling “1” if more than 50% of fresh produce items is not spoiled, high quality (e.g., no bruises, mold, etc.).

**Frozen Fruits and Vegetables**

- Any variety of frozen fruits without added sugars, fats, oils, or salt (i.e., sodium).
- Any variety of frozen vegetables without added sugars, fats, or oils. Vegetable(s) must be listed as the first ingredient. May be regular or lower in sodium.

**Packaged Fruits and Vegetables**

- Any variety of canned vegetables without added sugars, fats, or oils (e.g., do not count mashed potatoes). Vegetable(s) must be listed as the first ingredient. May be regular or lower in sodium. Includes yams and sauerkraut if they meet the requirements.
- Any variety of canned fruits (including applesauce, juice pack or water pack without added sugars, fats, oils, or salt (i.e., sodium). The fruit must be listed as the first ingredient.

**Dried Fruits and Vegetables**

- Any type of dried fruits without added sugars, fats, oils, or salt (i.e., sodium).
- Any type of dried vegetable without added sugars, fats, oils, or salt (i.e., sodium).

### Not Allowed

- herbs and spices
- creamed vegetables or vegetables with added sauces
- mixed vegetables containing noodles, nuts or sauce packets
- vegetable-grain (pasta or rice) mixtures
- fruit-nut mixtures
- breaded vegetables
- fruits and vegetables for purchase on salad bars
- peanuts or other nuts
- ornamental and decorative fruits and vegetables such as chili peppers on a string; garlic on a string
- gourds; painted pumpkins; fruit baskets and party vegetable trays
- decorative blossoms and flowers
- foods containing fruits such as blueberry muffins and other baked goods.
- home-canned and home-preserved fruits and vegetables.

**Allowable Juice**

- Any fruit and/or vegetable juice or juice blends (e.g., orange, grapefruit, apple, grape, pineapple, tomato, cran-apple)
- Fresh
- Single strength
- From concentrate
- Frozen
- Canned
- Shelf-stable

### Juice Nutrition Requirements

- 100% unsweetened pasteurized fruit and/or vegetable juice.
- Minimum of 30 milligrams of Vitamin C per 100 milliliters (about 3.38 oz) of juice, or 72 milligrams of Vitamin C per 8-fluid ounces (e.g., Vitamin C = 90% U.S. RDA for women per 6 fluid ounces juice).
- Vegetable juice may be regular or lower in sodium.

Not Allowed

- Fruit drinks
- Fruit-flavored ades
- Sodas
- Other beverages that are not 100% juice

## Protein and Protein Substitutes

*(Tool pages 13-16)*

For protein and protein substitutes, data collectors will document the availability of meat, poultry, fish, eggs, and non-animal proteins if it meets the nutritional requirements. Blank spaces are provided to document which type of beans, legumes, or peas are available. Meat products that are available in its/their whole form, pieces, and ground can be documented. For shelf-stable meat products and meat centered meals that list multiple types of meat, go by the first meat listed when deciding where to document on the tool. Please document whether fresh meat products are expired by circling “1” for “yes” and “0” for “no” under the column “Expired”?

**Meat, Poultry, and Fish*:**

• Whole, pieces, ground meat

- Includes beef, chicken, pork
- Examples: chicken breasts, ground beef, porkchop

Deli Meat

- Example: ham, turkey, salami

Bacon

- May be pork, turkey, beef etc.

Sausage

- May be pork, turkey, beef etc.

Hotdogs

- May be pork, turkey, beef etc.

Tofu

- Tofu-based alternatives to the meat/protein options listed above

**These are Non-WIC allowable food items*

### Meat-Centered Meals

Meat center meals are frozen items and combination dishes that you can heat and serve. Examples include potpies, pizza, chicken nuggets/sticks, burgers, and mixed pasta dishes like lasagna. You will also record whether there are vegetarian and tofu options available.

### Allowable Fish (Canned, jars, pouches, self-stable containers)

- Light tuna
- Salmon
- Sardines
- Mackerel (ONLY N. Atlantic Scoumber scombrus, Chub Pacific Scomber japonicas, or Jack Mackerel)

### Canned Fish Nutrition Requirements

- May be packed in water or oil.
- Pack may include bones or skin.
- May be regular or lower in sodium content.

### Allowable Nut and Seed Butters

- Creamy or chunky, regular or reduced fat, salted or unsalted
- Nut and seed butters

**Nut and Seed Butter Requirements**

- Must provide comparable nutritive value to peanut butter (i.e., protein and iron).

**Not Allowed**

- Peanut spreads.
- Added marshmallows, honey, jelly, chocolate, or similar ingredients.

### Allowable Mature Legumes (Dry Beans and Peas)

Any type of mature dry beans, peas, or lentils in dry-packaged or canned forms. Examples include but are not limited to black beans, black-eyed peas, garbanzo beans (chickpeas), great northern beans, white beans (navy and pea beans), kidney beans, mature lima beans ("butter beans"), fava and mung beans, pinto beans, soybeans, split peas, lentils and refried beans. Baked beans are only authorized for participants with limited cooking facilities. Circle “D” for dried or “C” for canned beans, peas, or lentils.

**Not Allowed**

- Soups
- Immature varieties of legumes, such as those used in canned green peas, green beans, snap beans, yellow beans, and wax beans.
- Baked beans with meat (e.g., beans and franks).

### Allowable Eggs

- Fresh shell Domestic hen’s eggs*: any size, white or brown shells.
- Dried egg mix
- Pasteurized liquid whole eggs

## Dairy and Dairy Substitutes

*(Tool pages 17-20)*

For dairy and dairy substitutes, data collectors will document the *availability* of cow’s milk in different forms, fat contents, and allowable milk substitutions. Data collectors should refer to the below nutritional requirements when deciding whether to count a food item and pay careful attention to the fat content, source (e.g., cow, soy), and form (e.g., cheese slices) listed on the instrument. For cheese, write in the type of cheese available (e.g., American).

### Allowable Types of Cow’s Milk

- Whole, reduced fat, low-fat, or nonfat
- Cultured milk
- Evaporated, Dry: Calcium-fortified milk
- Lactose-reduced and lactose-free milk
- Acidified milk
- Shelf stable

**Cow’s Milk Nutrition Requirements**

- Must be pasteurized.
- Must be unflavored.
- **For all fat varieties of milk: Must contain 400 International Units (2.5 micrograms) of Vitamin D per quart (100 IU per cup).**
  - **If vitamin D is not listed in micrograms or IU, document the largest and cheapest milk available and note the % Daily Value listed for Vitamin D.**
- **For only reduced fat, low-fat, or nonfat varieties (does not apply to whole milk): Must contain 2000 International Units of Vitamin A per quart (500 IU or 150 micrograms per cup)**
  - **If vitamin A is not listed in micrograms or IU, document the largest and cheapest milk available and note the % Daily Value listed for Vitamin A.**
  - **If vitamin A is not listed at all on the label (no longer a label requirement), document the largest and cheapest milk available and note that vitamin A amount could not be verified.**
- Nut milks are not allowed.
- Single serve units not allowed.
- Any plant-based milk alternative must meet the following to count as WIC-approved (otherwise, count as non-WIC approved):
  - Be fortified to meet the following nutrient levels per cup: 276 mg calcium, 8 g protein, 500 IU vitamin A, 100 IU vitamin D per, 24 mg magnesium, 222 mg phosphorus, 349 mg potassium, 0.44 mg riboflavin, and 1.1 mcg vitamin B12, in accordance with FDA fortification guidelines.
  - Must contain no more than 10 grams of added sugars per cup.
  - May be flavored or unflavored.

**Counts**

- When tallying WIC-approved and non-WIC approved milk varieties, count each different type (e.g., based on at content) and size (e.g., gallon, half-gallon). Single serve sizes not allowed.

**Notes**

1. State agencies may choose to authorize goat’s milk as a substitute for cow’s milk. WIC-authorized goat’s milk must meet the same requirements as cow’s milk.
2. Vitamin A is naturally found in the fat portion of milk, which is removed (all or partially) during the production of reduced-fat, low-fat, and fat-free milks. For this reason, Vitamin A fortification is required for these milks, but not whole milk. The nutrient requirement pertains to all authorized milks; for dried milk (i.e., powdered) and evaporated milk, vitamin requirements are per reconstituted quart.

### Allowable Cheese

- Pasteurized Processed American
- Brick
- Natural Cheddar
- Colby
- Monterey Jack
- Mozzarella: part skim or whole
- Muenster
- Provolone
- Swiss
- Plant-based cheese alternatives (e.g., soy)

* Note: Blends of approved cheeses, e.g., CoJack, are authorized.

### Cheese Nutrition Requirements

- Cow’s milk-based cheese must be domestic cheese made from 100% pasteurized milk.
- Cheeses that are labeled low, free, reduced, less or light in sodium, fat or cholesterol are allowed.
- Plant-based cheese alternatives must contain a minimum of 250 milligrams of calcium and 6.5 grams of protein per 1.5 ounces.

**Not Allowed**

- Cheese foods or spreads
- Imported cheeses
- Plant-based curd cheeses.

### Yogurt Nutrition Requirements

- Must be pasteurized
- May be plain or flavored.
- Yogurts fortified with vitamin A and other nutrients are allowed at the SA’s option.
- May be plant-based yogurt alternative
- Added sugars limit for cow’s milk-based yogurt is ≤ 16 grams per 8 ounces
- Added sugars limit for plant-based milk alternatives of ≤ 10 grams per 8 ounces.
- Plant-based milk must have a minimum of 250 milligrams of calcium and 6.5 grams of protein.
- All types of yogurts must contain a minimum of vitamin D specification of 106 IU (2.67 micrograms) per 8 oz (1 cup) of yogurt.

**Not Allowed**

- Yogurts sold with accompanying mix-in ingredients such as granola, candy pieces, honey, nuts and similar ingredients.
- Drinkable yogurts.

**In the example below, a serving is 113 grams (about 3.99 oz) and contains 11 grams of added sugar per serving.**

**First convert 113 grams (about 3.99 oz) to ounces [113/28.34952 = 3.98 ounces)**

**3.98 ounces goes into 8 ounces 2.01 times [8/3.98 = 2.01]**

**And so, 8 ounces of this yogurt would contain 22.11 grams of added sugar [2.01 x 11 grams of added sugar]. This product does not qualify.**


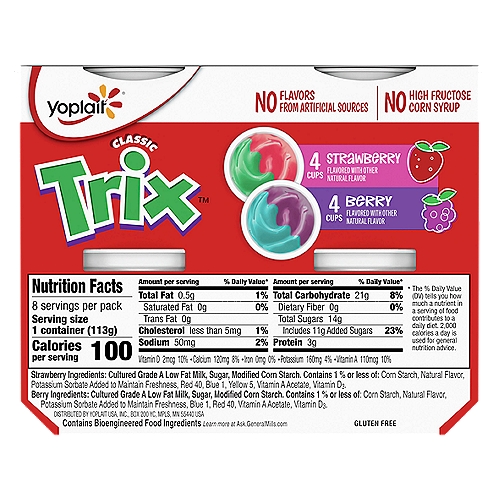


**Tofu Nutrition Requirements**

- Calcium-set tofu prepared with calcium salts (e.g., calcium sulfate), but may also contain other coagulants (i.e., magnesium chloride).
- May not contain added fats, sugars, oils or sodium.
- Minimum calcium specification of 100 milligrams per 100 grams (about 3.53 oz) of tofu.

**Plant-Based Beverage Nutrition Requirements**

- Must be fortified to meet the following nutrient levels per cup: 276 mg calcium, 8 g protein, 500 IU vitamin A, 100 IU vitamin D per, 24 mg magnesium, 222 mg phosphorus, 349 mg potassium, 0.44 mg riboflavin, and 1.1 mcg vitamin B12, in accordance with FDA fortification guidelines.
- Must contain no more than 10 grams of added sugar per cup.
- May be flavored or unflavored.

## Whole Grains

*(Tool pages 21-23)*

For whole grains, data collectors will document the availability of bread and cereals in different forms. Data collectors should refer to the below nutritional requirements when deciding whether to count a food item. The blank spaces are intended for listing the variety of ready-to-eat cereal available as well as additional varieties of whole grains that may not be listed such as bulgur, barley, etc.

### Allowable Whole Wheat Bread/Whole Grain Bread/Other Whole Grains

- Whole wheat bread, buns and rolls
- Whole grain bread, buns and rolls
- Whole grain options (may be instant-, quick-, or regular-cooking): brown rice, wild rice, quinoa, bulgur, oats, and whole-grain barley, millet, triticale, amaranth, cornmeal (including blue), corn masa flour, whole wheat macaroni (pasta) products, whole wheat bread products (i.e., pita, English muffin, bagels, naan), soft corn or whole wheat tortillas, buckwheat, teff, kamut, or sorghum, wheat berries, and other intact whole grains (e.g., red rice, black rice, freekeh, spelt, farro, etc.).

### Whole Grain Nutrition Requirements

- For whole wheat bread (includes whole grain buns and rolls), “whole wheat flour” and/or “bromated whole wheat flour” must be the **only** flours listed in the ingredient list.
- For whole grain bread (includes whole grain buns and rolls), “whole grain” must be the primary ingredient by weight in all whole grain bread products.
- Whole grain bread(s) must contain at least 50 percent whole grains.
- Whole wheat macaroni products must have no added sugars, fats, oils, or salt (i.e., sodium). ‘‘Whole wheat flour’’ and/or ‘‘whole durum wheat flour’’ must be the only flours listed in the ingredient list. If these requirements are met, other shapes and sizes are allowed (e.g., whole wheat rotini and whole wheat penne).
- Corn tortillas made from ground masa flour (corn flour) using traditional processing methods are allowed. Examples of primary ingredients meeting the WIC-eligibility criteria include whole corn, corn (masa), whole ground corn, corn masa flour, masa harina, and white corn flour.

**Not Allowed**

- Whole grain options may not contain added fats, sugars, oils, or sodium – e.g., macaroni and cheese, pasta with sauce, cheesy rice.

### Allowable Breakfast Cereal

- Ready-to-eat (e.g., corn flakes, bran flakes)
- Instant and regular hot cereals (e.g., oatmeal, grits, cream of wheat)

### Breakfast Cereal Nutrition Requirements

- Must contain a minimum of 28 milligrams of Iron per 100 grams (about 3.53 oz) of dry cereal (e.g., iron = 45% U.S. RDA for adults per 1-ounce dry cereal).
- Must contain ≤ 21.2 grams of added sugar per 100 grams (≤ 6 g per 1 oz).
- “Whole grain” must be the primary ingredient by weight in all whole grain cereal products.

**In the example below, a serving is 29 grams and contains 6 grams of sugar per serving.**

**At 29 grams per serving, a serving goes into 100 grams (about 3.53 oz) 3.45 times (100 grams /29 grams = 3.45) and so 100 grams of this cereal would contain 3.45 x 6 grams of sugar = 20.68 grams which is under the limit of 21.2. Therefore, this cereal would qualify**.


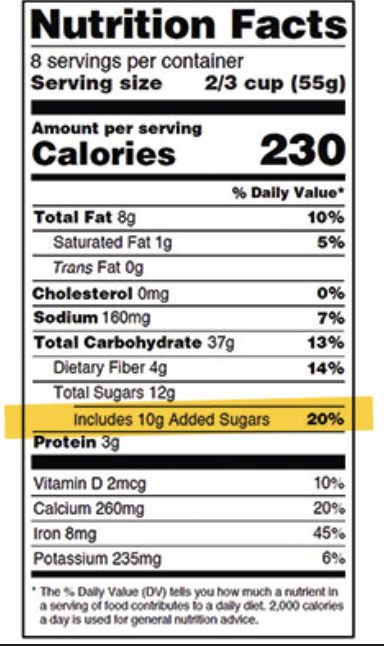


## Infant Formula and Food

*(Tool pages 24-28)*

For infant food and formula, data collectors will document the availability of infant formulas, nutritionals, and food. Data collectors should refer to the below nutritional requirements when deciding whether to count a food item. Data collectors should pay careful attention to the milk source (e.g., cow, soy), and form (e.g., powder, concentrated) for infant formulas listed on the instrument. There are rows provided to count up to 3 brands (A, B, and C) for cow’s milk-based infant formulas and up to 2 brands (D and E) for soymilk based infant formulas. This is the only food category that data collectors will document the brand of formula.

Exempt infant formulas include any formula that is described as “hypoallergenic,” specialized,” or intended for premature infants. Nutritionals are medical foods prescribed to children for a condition such as failure-to-thrive. Blank spaces are provided to document different types of exempt infant formulas and nutritionals. *For example, for the exempt infant formula Nutramigen, a data collector would write “hypoallergenic” in a blank space and the brand “Nutramigen” in the brand column. For the nutritional Pediasure with Fiber, a data collector would write “weight grain nutritional drink with fiber” in a blank space and the brand “Pediasure” in the brand column.*

Blank spaces are provided to document the different flavors of infant fruits, vegetables, and meat. Please document whether infant formula and nutritional products are expired by circling “1” for “yes” and “0” for “no” under the column “Expired”?

## Allowable Infant Formula

- Concentrated liquid
- Powdered
- Ready-to-feed (RTF)

## Infant Formula Nutrition Requirements

- Nutritionally complete infant formula does not require the addition of any ingredients other than water prior to being served in a liquid state.
- Be designed for enteral digestion via oral or tube feeding.
- Provide at least 10 mg iron per liter (at least 1.5 mg iron/100 kilocalories) at standard dilution.
- Provide at least 67 kilocalories per 100 milliliters (approximately 20 kilocalories per fluid ounce) at standard dilution.

## Allowable Exempt Infant Formula (Hypoallergenic/Specialized/Premature)

- Concentrated liquid
- Powdered
- Ready-to-feed (RTF) or ready-to use (RTU)

## Exempt Infant Formula Nutrition Requirements

- Be designed for enteral digestion via oral or tube feeding.

## Allowable Nutritionals

- Concentrated liquid
- Powdered
- Ready-to-feed (RTF) or ready-to use (RTU)

## Nutritionals Nutrition Requirements

- Intended for use as an oral feeding and may not be a conventional food; formulas administered through a nasogastric tube may be substituted.
- Must serve the purpose of a food, meal or diet (may be nutritionally complete or incomplete) and provide a source of calories and one or more nutrients (may be nutritionally complete or incomplete).

**Not Allowed**

- Formulas used solely for the purpose of enhancing nutrient intake or managing body weight addressing picky eaters or used for a condition other than a qualifying condition (e.g., vitamin pills, weight control products)
- Medicines or drugs
- Hyperalimentation feedings (nourishment administered through a vein)
- Enzymes, herbs, or botanicals
- Oral rehydration fluids or electrolyte solutions
- Flavoring or thickening agents
- Feeding utensils or devices (e.g., feeding tubes, bags, pumps) designed to administer a WIC-eligible formula
- Sports or breakfast drinks


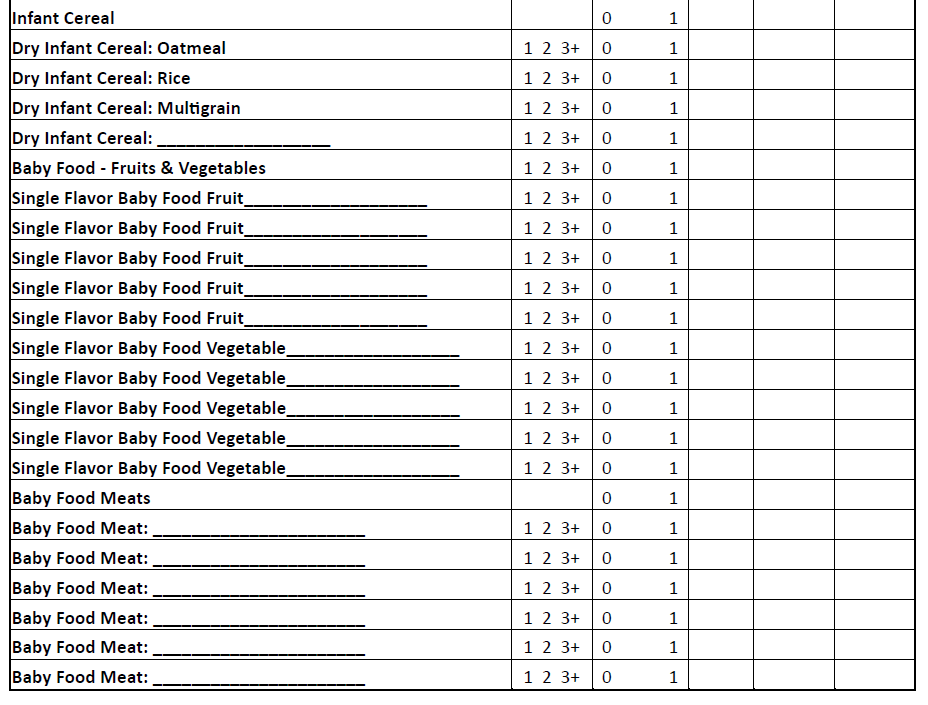


## Allowable Infant Cereal

- Any plain, dry infant cereal (e.g., rice, barley, mixed grain)

## Infant Cereal Nutrition Requirements

- Minimum of 45 milligrams of Iron per 100 grams of dry cereal.

**Not Allowed**

- Infant cereal with added infant formula, milk, fruit, or other non-cereal ingredients.

## Allowable Infant Food - Fruits and Vegetables

- Any variety of single ingredient commercial infant food fruit without added sugars, starches, or salt (e.g., sodium). Texture may range from strained through diced. The fruit must be listed as the first ingredient.
- Any variety of single ingredient commercial infant food vegetables without added sugars, starches, or salt (e.g., sodium). Texture may range from strained through diced. The vegetable must be listed as the first ingredient.
- Combinations of single ingredients (e.g., peas-carrots and apple-banana) are allowed.
- Pouches are allowed.

**Not Allowed**

- Mixtures with cereal or infant food desserts (e.g., peach cobbler).

### Allowable Infant Food - Meat

- Any variety of commercial infant food, meat or poultry, as a single major ingredient, with added broth or gravy. Texture may range from pureed through diced.

**Not Allowed**

- Added sugars or salt (e.g., sodium).
- Infant food combinations (e.g., meat and vegetables) or dinners (e.g., spaghetti and meatballs).

# Measuring Shelf Space

*(Tool page 29)*

This is a one-dimensional measurement referring to the length of the shelf or cooler/refrigerator. To measure linear shelf space:

- Use a measure app on your smartphone to record the length of the shelf to the nearest inch.
- Consider a row of hooks/hanging items to be one shelf.
- For baskets or other round/oval displays, measure the diameter at the widest point. Baskets > 50% full can assumed to be full.
- It is common for aisles to have multiple shelves stacked vertically; to save time, record the length of the measured shelf by the number of shelves (e.g., 4 x 36 in, 6 x 24 in) in the worksheet. Note that most shelves come in standard sizes (e.g., 24, 36, or 48 in).
- Do NOT count empty shelves.
- For island displays of soda (i.e., cases stacked on top of one another on the floor without separate shelving), treat as 1 shelf and only measure the length.
- Additional Tips:
- Start at one end of the store and work your way through the aisles methodically. Do all the coolers, impulse buy sections, seasonal promotion areas, and ends of aisles first so you don't forget what you've already counted.
- Add up all areas to provide the total value in inches; then round to the nearest foot.
- If in a pair, you can have one person measure the shelves and call out measurements and multipliers (e.g., 4 by 48), while the other person writes down the numbers.
- Practice measuring with the app before you begin data collection. We also suggest that you ensure that your app is reliable by measuring a 12” ruler.
- Complete the shelf space measurements last, after the remainder of the tool is completed
- Depending on the app you are using, there may be a feature to take a screen shot of each measurement which can serve as means of validating your measurement later after your visit is complete.

**Measure all the following food & beverage categories:**

- **Fresh Fruit:** Include all fresh fruit.
- **Fresh vegetables:** Include all fresh vegetables.
- **Canned Fruit:** Include all canned fruit, including those that are packed in syrup.
- **Canned vegetables:** Include all canned vegetables, including those that are low in sodium.
- **Frozen Fruit:** Include all frozen fruit, including those with added sugar.
- **Frozen vegetables:** Include all frozen vegetables.
- **Sugar-sweetened beverages:** Includes soda, sports drinks, energy drinks, non 100% juice, or sweetened coffee drinks. INCLUDE all beverages EXCEPT: Plain water (still & carbonated water with no added caloric/non-caloric sweeteners); 100% fruit juice (includes coconut water); Plain milk (cow’s milk and milk substitutes with no added sweeteners); and Alcohol.
- **Bottled water:** INCLUDE only plain water: still & carbonated water with no added caloric/non-caloric sweeteners.
- **Packaged salty snacks:** Include chips, popped and/or flavored popcorn, pretzels, and salted meat snacks such as beef jerky. Count Cheez-Its, Combos, crack sandwiches, and other similar processed salty snacks. Do NOT include nuts, rice cakes or crackers.
- **Packaged sweet snacks:** Include chocolates, other candy, cookies, snack cakes, brownies, pastries/ pies, donuts, sweet rolls and similar sweet processed (shelf stable) foods. Do NOT include refrigerated/ frozen sweet snacks, like ice cream and other frozen desserts; cereal and nutrition bars (e.g., Nutri-Grain bar, PowerBar); and dried fruit snacks.

# Non-Food Items

*(Tool page 30)*

Data collectors will indicate whether each non-food item is available or not. For cleaning products (laundry detergent, window cleaner, bleach wipes, and toilet paper) document the lowest priced product (no sales) that is the largest size. For over-the-counter medicines document the lowest priced product (no sales) that is the largest size. Document the types of different medications available. Be sure to document the size, price, and brand.

**Appendix B NEMS-DS**

**The Nutrition Environment Measures Survey for Dollar Stores**

**Data Collection Information**

Date of visit:_______________

Time of visit:_______________

Data Collector Names:_____________________________________________________________

Store Address:___________________________________________________________________

Store Hours of Operation:__________________________________________________________

Completion Tips

Remember to follow the tips below to decrease the data cleaning time for data entry.

1. If you are completing a paper form, WRITE LEGIBLY! This form will likely be entered by someone else, and your work must be legible to them.
2. Check your work.
3. Use the correct line/check box.
4. Write your comments and notes on the sections provided. Only use the margins as a last resort.
5. Do not cross through any individual items or sections.
6. If you are using a paper form, please USE PENCIL. Erase any stray marks you make.
7. For data on specific products, only fill in subsequent information if the product is available.
8. Complete only 1 assessment form per store; when completing in teams, one data collector completes the form while the other data collector assists.
9. Check instructions and the manual to ensure that you follow the protocol.
10. Call your site preceptor if you have any questions.

|  | **CIRCLE ONE** | **CONDITIONS OUTSIDE STORE** | **NO** | **YES** | **CONDITIONS INSIDE STORE** | **NO** | **YES** |
| --- | --- | --- | --- | --- | --- | --- | --- |
| **Store brand** |  | Parking | 0 | 1 | Trash/debris | 0 | 1 |
| **Dollar Tree** | 1 | Trash/debris | 0 | 1 | Cluttered floors | 0 | 1 |
| **Family Dollar** | 2 | Broken glass | 0 | 1 | Foul odor | 0 | 1 |
| **Dollar General** | 3 | Graffiti | 0 | 1 | Panhandling | 0 | 1 |
| **Combo Store (Family Dollar/Dollar Tree)** | 4 | Loitering | 0 | 1 | High shelving/can't see across store | 0 | 1 |
| **99 Cent Only Store** | 5 | Shopping cart rails | 0 | 1 | Security Cameras | 0 | 1 |
| **Other: _________** | 6 | Panhandling | 0 | 1 | Security Personnel | 0 | 1 |
|  |  | Bus stop nearby | 0 | 1 | Other: ___ | 0 | 1 |
|  |  | Landscaping | 0 | 1 | Shopping cart rails | 0 | 1 |
|  |  | Security Cameras | 0 | 1 | **FEDERAL PROGRAMS** |  |  |
|  |  | Security bars on windows/doors | 0 | 1 | Does the store accept EBT/SNAP? | 0 | 1 |
|  |  | Other: |  |  | Does the store accept WIC? | 0 | 1 |

| **Promoted Items** | | |
| --- | --- | --- |
|  | YES | NO |
| Can fresh produce (fruits and/or vegetables) be seen from the front entrance? | [   ] | [   ] |
| Can frozen produce (fruits and/or vegetables) be seen from the front entrance? | [   ] | [   ] |
| Can canned produce (fruits and/or vegetables) be seen from the front entrance? | [   ] | [   ] |
| Can salty snacks be seen from the front entrance? | [   ] | [   ] |
| Can sweet snacks be seen from the front entrance? | [   ] | [   ] |
| Can sugar sweetened beverages be seen from the front entrance? | [   ] | [   ] |
| Can bottled water be seen from the front entrance? | [   ] | [   ] |

Number of checkouts (include all checkouts: e-checkouts/employee stationed/no employee stationed)

| **Checkout Items** | | |
| --- | --- | --- |
|  | YES | NO |
| Are fresh produce (fruits and/or vegetables) available at checkout? | [   ] | [   ] |
| Are frozen produce (fruits and/or vegetables) available at checkout? | [   ] | [   ] |
| Are canned produce (fruits and/or vegetables) available at checkout? | [   ] | [   ] |
| Are salty snacks be seen from the front entrance available at checkout? | [   ] | [   ] |
| Are sweet snacks be seen from the front entrance available at checkout? | [   ] | [   ] |
| Are sugar sweetened beverages be seen from the front entrance available at checkout? | [   ] | [   ] |
| Are bottled water be seen from the front entrance available at checkout? | [   ] | [   ] |

**Fruits & Vegetables: FRESH (Enter other items in blank boxes)**

| **Fruits** | **Amount** | **Acceptability** | **Price (per item/lb.)** | **Vegetables** | **Amount** | **Acceptability** | **Price (per item/lb.)** |
| --- | --- | --- | --- | --- | --- | --- | --- |
| **Bananas** | 0 1 2 3+ | 0 1 |  | **Potatoes** | 0 1 2 3+ | 0 1 |  |
| **Apples** | 0 1 2 3+ | 0 1 |  | **Onions** | 0 1 2 3+ | 0 1 |  |
| **Oranges** | 0 1 2 3+ | 0 1 |  | **Celery** | 0 1 2 3+ | 0 1 |  |
| **Lemons** | 0 1 2 3+ | 0 1 |  | **Leafy lettuce (any kind bagged or non-bagged)** | 0 1 2 3+ | 0 1 |  |
| **Strawberries** | 0 1 2 3+ | 0 1 |  | **Peppers** | 0 1 2 3+ | 0 1 |  |
| **Tomatoes** | 0 1 2 3+ | 0 1 |  | **Cucumbers** | 0 1 2 3+ | 0 1 |  |
|  | 0 1 2 3+ | 0 1 |  |  | 0 1 2 3+ | 0 1 |  |
|  | 0 1 2 3+ | 0 1 |  |  | 0 1 2 3+ | 0 1 |  |
|  | 0 1 2 3+ | 0 1 |  |  | 0 1 2 3+ | 0 1 |  |
|  | 0 1 2 3+ | 0 1 |  |  | 0 1 2 3+ | 0 1 |  |
|  | 0 1 2 3+ | 0 1 |  |  | 0 1 2 3+ | 0 1 |  |
|  | 0 1 2 3+ | 0 1 |  |  | 0 1 2 3+ | 0 1 |  |
| **Total Number of Fresh, WIC-approved Fruit Varieties:** | **Total Number of Fresh Fruit Varieties:** | | **Total Number of Fresh, WIC-approved Vegetable Varieties:** | | **Total Number of Fresh Vegetable Varieties:** | | |

**FRESH JUICE: FRUIT AND VEGETABLE (Refrigerated)**

**Location (Fresh/Perishable Fruit Juice): SD H EL L Other_____________**

**Location (Fresh/Perishable Vegetable Juice): SD H EL L Other_____________**

| **Fruit/Vegetable** | **Type (circle one)** | **Size (circle one)** | **Amount** | **Price** |
| --- | --- | --- | --- | --- |
| **Example: Apples** | Fruit Vegetable | 11.5oz 12oz 48oz 64oz Other:___ | 0 1 2 3+ |  |
|  | Fruit Vegetable | 11.5oz 12oz 48oz 64oz Other:___ | 0 1 2 3+ |  |
|  | Fruit Vegetable | 11.5oz 12oz 48oz 64oz Other:___ | 0 1 2 3+ |  |
|  | Fruit Vegetable | 11.5oz 12oz 48oz 64oz Other:___ | 0 1 2 3+ |  |
|  | Fruit Vegetable | 11.5oz 12oz 48oz 64oz Other:___ | 0 1 2 3+ |  |
|  | Fruit Vegetable | 11.5oz 12oz 48oz 64oz Other:___ | 0 1 2 3+ |  |
|  | Fruit Vegetable | 11.5oz 12oz 48oz 64oz Other:___ | 0 1 2 3+ |  |
|  | Fruit Vegetable | 11.5oz 12oz 48oz 64oz Other:___ | 0 1 2 3+ |  |
| **Total Number of WIC-approved fresh/perishable Produce Juice Varieties:** | | | **Total Number of fresh/ perishable Produce Juice Varieties:** | |

**Fruits: FROZEN & FROZEN Juice Concentrate**

**Location (Frozen Fruits): SD H EL L Other_____________**

**Location (Frozen Juice Concentrate): SD H EL L Other_____________**

| **Fruit** | **Amount** | **Size (oz./lbs.)** | **Price (per bag/container)** | **Frozen Concentrate** | **Size** | **Amount** | **Price (per bag/container)** |
| --- | --- | --- | --- | --- | --- | --- | --- |
|  | 0 1 2 3+ |  |  | Concentrate | 11.5oz 12oz Other:__ | 0 1 2 3+ |  |
|  | 0 1 2 3+ |  |  | Concentrate | 11.5oz 12oz Other:__ | 0 1 2 3+ |  |
|  | 0 1 2 3+ |  |  | Concentrate | 11.5oz 12oz Other:__ | 0 1 2 3+ |  |
|  | 0 1 2 3+ |  |  | Concentrate | 11.5oz 12oz Other:__ | 0 1 2 3+ |  |
|  | 0 1 2 3+ |  |  | Concentrate | 11.5oz 12oz Other:__ | 0 1 2 3+ |  |
|  | 0 1 2 3+ |  |  | Concentrate | 11.5oz 12oz Other:__ | 0 1 2 3+ |  |
|  | 0 1 2 3+ |  |  | Concentrate | 11.5oz 12oz Other:__ | 0 1 2 3+ |  |
|  | 0 1 2 3+ |  |  | Concentrate | 11.5oz 12oz Other:__ | 0 1 2 3+ |  |
|  | 0 1 2 3+ |  |  | Concentrate | 11.5oz 12oz Other:__ | 0 1 2 3+ |  |
|  | 0 1 2 3+ |  |  | Concentrate | 11.5oz 12oz Other:__ | 0 1 2 3+ |  |
| **Total Number of Frozen, WIC-approved Fruit and Fruit Juice Varieties:** | | | **Total Number of Frozen Fruit and Fruit Juice Varieties:** | | | | |

**Vegetables: FROZEN**

**Location Vegetables (Frozen): SD H EL L Other_____________**

| **Vegetable** | **Amount** | **Size (oz./lbs.)** | **Price (per bag/container)** |
| --- | --- | --- | --- |
|  | 0 1 2 3+ |  |  |
|  | 0 1 2 3+ |  |  |
|  | 0 1 2 3+ |  |  |
|  | 0 1 2 3+ |  |  |
|  | 0 1 2 3+ |  |  |
|  | 0 1 2 3+ |  |  |
|  | 0 1 2 3+ |  |  |
|  | 0 1 2 3+ |  |  |
|  | 0 1 2 3+ |  |  |
| **Total Number of Frozen, WIC-approved Vegetable Varieties:** | | **Total Number of Frozen Vegetable Varieties:** | |

**Grains and Tortillas: FRESH and FROZEN**

| **Grains** | **Type** | **Amount** | **Expired? (1 for Yes)** | **Size** | **Type** | **Amount** | **Expired? (1 for Yes)** |
| --- | --- | --- | --- | --- | --- | --- | --- |
| Whole Grain Tortilla: Corn | Fresh | 0  1  2  3+ | 0             1 | 16oz  Other:__ | Frozen | 0  1  2  3+ | ​​0             1 |
| Whole Grain Tortilla: Wheat | Fresh | 0  1  2  3+ | 0             1 | 16oz  Other:__ | Frozen | 0  1  2  3+ | ​​0             1 |
| Whole Grain Bread | Fresh | 0  1  2  3+ | 0             1 | 16oz  Other:__ | Frozen | 0  1  2  3+ | ​​0             1 |
| Whole Grain Buns | Fresh | 0  1  2  3+ | 0             1 | 16oz  Other:__ | Frozen | 0  1  2  3+ | ​​0             1 |
| Whole Grain Rolls | Fresh | 0  1  2  3+ | 0             1 | 16oz  Other:__ | Frozen | 0  1  2  3+ | ​​0             1 |
| Whole Grain Pasta | Fresh | 0  1  2  3+ | 0             1 | 16oz  Other:__ | Frozen | 0  1  2  3+ | ​​0             1 |
|  | Fresh | 0  1  2  3+ | 0             1 | 16oz  Other:__ | Frozen | 0  1  2  3+ | ​​0             1 |
|  | Fresh | 0  1  2  3+ | 0             1 | 16oz  Other:__ | Frozen | 0  1  2  3+ | ​​0             1 |
|  | Fresh | 0  1  2  3+ | 0             1 | 16oz  Other:__ | Frozen | 0  1  2  3+ | ​​0             1 |
| **Total Number of WIC-approved Fresh (Refrigerated) Tortilla/Breads/Grains Varieties:** | | **Total Number of Fresh (Refrigerated) Tortilla/Breads/Grains Varieties:** | | **Total Number of WIC-approved FROZEN Tortilla/Breads/Grains Varieties:** | | **Total Number of FROZEN Tortilla/Breads/Grains Varieties:** | |

**Dairy and Dairy Substitutes**

|  | **Perishable** | **Amount** | **Expired? (1 for Yes)** | **Price** | **Size** | **Shelf-Stable** | **Amount** | **Expired? (1 for Yes)** |
| --- | --- | --- | --- | --- | --- | --- | --- | --- |
| Whole Cow's Milk | Fresh | 0 1 2 3+ | 0 1 |  | Gallon  Half gallon  Quart |  |  |  |
| Whole Cow's Milk |  |  |  |  |  | Shelf-stable, Liquid | 0 1 2 3+ | 0 1 |
| Whole Cow's Milk |  |  |  |  |  | Shelf-stable, Powdered | 0 1 2 3+ | 0 1 |
| 1% or Skim Cow's Milk | Fresh | 0 1 2 3+ | 0 1 |  | Gallon  Half gallon  Quart |  |  |  |
| 1% or Skim Cow's Milk |  |  |  |  |  | Shelf-stable, Liquid | 0 1 2 3+ | 0 1 |
| 1% or Skim Cow's Milk |  |  |  |  |  | Shelf-stable, Powdered | 0 1 2 3+ | 0 1 |
|  | **Perishable** | **Amount** | **Expired? (1 for Yes)** | **Price** | **Size** | **Shelf-Stable** | **Amount** | **Expired? (1 for Yes)** |
| 2% Cow's Milk | Fresh | 0 1 2 3+ | 0 1 |  | Gallon  Half gallon  Quart |  |  |  |
| 2% Cow's Milk |  |  |  |  |  | Shelf-stable, Liquid | 0 1 2 3+ | 0 1 |
| 2% Cow's Milk |  |  |  |  |  | Shelf-stable, Powdered | 0 1 2 3+ | 0 1 |
| Non-fat Cow's Milk | Fresh | 0 1 2 3+ | 0 1 |  | Gallon  Half gallon  Quart |  |  |  |
| Non-fat Cow's Milk |  |  |  |  |  | Shelf-stable, Liquid | 0 1 2 3+ | 0 1 |
| Non-fat Cow's Milk |  |  |  |  |  | Shelf-stable, Powdered | 0 1 2 3+ | 0 1 |
| **Total Number of WIC-approved Fresh Cow’s Milk Varieties:** | | **Total Number of Fresh Cow’s Milk Varieties:** | |  | **Total Number of WIC-approved Shelf-Stable Cow’s Milk Varieties:** | | **Total Number of Shelf-Stable Cow’s Milk Varieties:** | |
| ***Milk Substitutions:*** |  |  |  |  |  |  |  |  |
| Milk (Goat) | Fresh | 0 1 2 3+ | 0 1 |  |  |  |  |  |
| Fortified Soy-based Beverage | Fresh | 0 1 2 3+ | 0 1 |  |  | Shelf-stable, Liquid | 0 1 2 3+ | 0 1 |
| Lactose-free milk | Fresh | 0 1 2 3+ | 0 1 |  |  | Shelf-stable, Liquid | 0 1 2 3+ | 0 1 |
| **Total Number of WIC-approved Fresh Dairy Sub Varieties:** | | **Total Number of Fresh Dairy Sub Varieties:** | | | **Total Number of WIC-approved Shelf-Stable Dairy Sub Varieties:** | | **Total Number of Shelf-Stable Dairy Sub Varieties:** | |
| Whole Milk Yogurt (Cow) | Fresh | 0 1 2 3+ | 0 1 |  | 32oz  64oz  Other:__ |  |  |  |
| Lower fat Yogurts (includes 2%, low fat, and nonfat) (cow) | Fresh | 0 1 2 3+ | 0 1 |  | 32oz  64oz  Other:__ |  |  |  |
| Yogurt (Plant-based) | Fresh | 0 1 2 3+ | 0 1 |  | 32oz  64oz  Other:__ |  |  |  |
| **Total Number of WIC-approved Fresh Yogurt Varieties:** | | **Total Number of Fresh Yogurt Varieties:** | | | |  |  |  |
|  | **Perishable** | **Amount** | **Expired? (1 for Yes)** | **Price** | **Size** |  |  |  |
| Cheese (Cow, Blocks) ___________ | Fresh | 0 1 2 3+ | 0 1 |  | 16oz. 8oz.  Other: ___ |  |  |  |
| Cheese (Cow, Blocks) ___________ | Fresh | 0 1 2 3+ | 0 1 |  | 16oz. 8oz.  Other: ___ |  |  |  |
| Cheese (Cow, Blocks) ____________ | Fresh | 0 1 2 3+ | 0 1 |  | 16oz. 8oz.  Other: ___ |  |  |  |
| Cheese (Cow, Slices) ____________ | Fresh | 0 1 2 3+ | 0 1 |  | 16oz. 8oz.  Other: ___ |  |  |  |
| Cheese (Cow, Slices) ____________ | Fresh | 0 1 2 3+ | 0 1 |  | 16oz. 8oz.  Other: ___ |  |  |  |
| Cheese (Cow, Slices) ____________ | Fresh | 0 1 2 3+ | 0 1 |  | 16oz. 8oz.  Other: ___ |  |  |  |
| Cheese (Cow, Shredded) ____________ | Fresh | 0 1 2 3+ | 0 1 |  | 16oz. 8oz.  Other: ___ |  |  |  |
| Cheese (Cow, Shredded) ____________ | Fresh | 0 1 2 3+ | 0 1 |  | 16oz. 8oz.  Other: ___ |  |  |  |
| Cheese (Cow, Stick) ____________ | Fresh | 0 1 2 3+ | 0 1 |  | 16oz. 8oz.  Other: ___ |  |  |  |
| Cheese (Plant-based) | Fresh | 0 1 2 3+ | 0 1 |  | 16oz. 8oz.  Other: ___ |  |  |  |
| **Number of WIC-approved Fresh Cheese Varieties:** | | **Total Number of Fresh Cheese Varieties:** | | |  |  |  |  |
| Tofu (Soy-based) | Fresh | 0 1 2 3+ | 0 1 |  |  |  |  |  |
| **Total Number of WIC-approved Tofu Varieties:** |  | **Total Number of Tofu Varieties:** |  |  |  |  |  |  |

**Proteins/Protein Substitutes (FRESH/FROZEN)**

| **Meat** | **Perishable** | **Amount** | **Expired?** | **Price** | **Size** | **Perishable** | **Amount** | **Size** |
| --- | --- | --- | --- | --- | --- | --- | --- | --- |
| Whole, pieces, ground meat (beef, chicken, pork; etc.) | Fresh | 0 1 2 3+ | 0 1 |  |  | Frozen | 0 1 2 3+ |  |
| Deli Meat (e.g., ham, turkey, salami) | Fresh | 0 1 2 3+ | 0 1 |  |  | Frozen | 0 1 2 3+ |  |
| Bacon (e.g., pork, turkey) | Fresh | 0 1 2 3+ | 0 1 |  |  | Frozen | 0 1 2 3+ |  |
| Sausage (e.g., pork, beef) | Fresh | 0 1 2 3+ | 0 1 |  |  | Frozen | 0 1 2 3+ |  |
| Hot Dogs (e.g., pork, beef) | Fresh | 0 1 2 3+ | 0 1 |  |  | Frozen | 0 1 2 3+ |  |
| Other (enter) | Fresh | 0 1 2 3+ | 0 1 |  |  | Frozen | 0 1 2 3+ |  |
| Other (enter) | Fresh | 0 1 2 3+ | 0 1 |  |  | Frozen | 0 1 2 3+ |  |
| Other (enter) | Fresh | 0 1 2 3+ | 0 1 |  |  | Frozen | 0 1 2 3+ |  |
| **Other** |  |  |  |  |  |  |  |  |
| **Eggs** | Fresh | 0 1 2 3+ | 0 1 |  |  |  |  |  |
| **Total Number of WIC-approved eggs:** | **Total Number of eggs:** | |  |  |  |  |  |  |

| **Meat Centered Meals** | **Meal** | **Amount** |
| --- | --- | --- |
| Beef/pork | Frozen pot pie | 0 1 2 3+ |
|  | Frozen mixed pasta dish (e.g., lasagna) | 0 1 2 3+ |
|  | Frozen pizza | 0 1 2 3+ |
|  | Burger | 0 1 2 3+ |
|  | Other, mixed dish_____________________ | 0 1 2 3+ |
| Chicken/turkey | Frozen pot pie | 0 1 2 3+ |
|  | Frozen mixed pasta dish (e.g., lasagna) | 0 1 2 3+ |
|  | Frozen Pizza | 0 1 2 3+ |
|  | Frozen Nuggets/sticks | 0 1 2 3+ |
|  | Burger | 0 1 2 3+ |
|  | Other, mixed dish_____________________ | 0 1 2 3+ |
| Tofu (meat substitute) | Frozen Tofu Meal | 0 1 2 3+ |
| Other frozen vegetarian option | (e.g., cheese pizza, macaroni and cheese, bean and cheese burrito) | 0 1 2 3+ |

**Proteins/Protein Substitutes (SHELF STABLE)**

|  | **Shelf-Stable** | **Amount** | **Price** | **Size** |
| --- | --- | --- | --- | --- |
| **Fish** |  | 0 1 2 3+ |  |  |
| Tuna | Canned | 0 1 2 3+ |  |  |
| Salmon | Canned | 0 1 2 3+ |  |  |
| Sardines | Canned | 0 1 2 3+ |  |  |
| Mackerel | Canned | 0 1 2 3+ |  |  |
| **Total Number of WIC-approved canned fish varieties:** | | **Total Number of packaged fish varieties:** | |  |
| **Beans/Legumes** |  | 0 1 2 3+ |  |  |
| Beans _______ | D C | 0 1 2 3+ |  |  |
| Beans________ | D C | 0 1 2 3+ |  |  |
| Beans ________ | D C | 0 1 2 3+ |  |  |
| Beans ________ | D C | 0 1 2 3+ |  |  |
| Beans ________ | D C | 0 1 2 3+ |  |  |
| Beans _______ | D C | 0 1 2 3+ |  |  |
| Peas________ | D C | 0 1 2 3+ |  |  |
| Peas________ | D C | 0 1 2 3+ |  |  |
| Peas________ | D C | 0 1 2 3+ |  |  |
| Lentils________ | D C | 0 1 2 3+ |  |  |
| Lentils________ | D C | 0 1 2 3+ |  |  |
| Lentils________ | D C | 0 1 2 3+ |  |  |
| **Number of WIC-approved bean/legume varieties:** |  | **Total Number of bean/legume varieties:** |  |  |
| Peanut Butter | Shelf Stable | 0 1 2 3+ | $ | 16 oz  18 oz  Other: _____ |
| **Total Number of WIC-approved peanut butter varieties:** | | **Total Number of peanut butter varieties:** | | |

**Fruit: Juice and Shelf-Stable**

**Location (Juice): SD H EL L Other_____________**

**Location (Canned): SD H EL L Other_____________**

**Location (Dried): SD H EL L Other_____________**

| **Fruits** | **Shelf-Stable (circle one)** | **Size (circle one)** | **Amount** | **Price** | **Shelf-Stable** | **Amount** | **Shelf-Stable** | **Amount** |
| --- | --- | --- | --- | --- | --- | --- | --- | --- |
| **Example: Apples** | Juice or Concentrate | 11.5oz 12oz  48oz  64oz  Other:___ | 0 1 2 3+ |  | Dried | 0 1 2 3+ | Canned | 0 1 2 3+ |
|  | Juice or Concentrate | 11.5oz 12oz  48oz  64oz  Other:___ | 0 1 2 3+ |  | Dried | 0 1 2 3+ | Canned | 0 1 2 3+ |
|  | Juice or Concentrate | 11.5oz 12oz  48oz  64oz  Other:___ | 0 1 2 3+ |  | Dried | 0 1 2 3+ | Canned | 0 1 2 3+ |
|  | Juice or Concentrate | 11.5oz 12oz  48oz  64oz  Other:___ | 0 1 2 3+ |  | Dried | 0 1 2 3+ | Canned | 0 1 2 3+ |
|  | Juice or Concentrate | 11.5oz 12oz  48oz  64oz  Other:___ | 0 1 2 3+ |  | Dried | 0 1 2 3+ | Canned | 0 1 2 3+ |
|  | Juice or Concentrate | 11.5oz 12oz  48oz  64oz  Other:___ | 0 1 2 3+ |  | Dried | 0 1 2 3+ | Canned | 0 1 2 3+ |
|  | Juice or Concentrate | 11.5oz 12oz  48oz  64oz  Other:___ | 0 1 2 3+ |  | Dried | 0 1 2 3+ | Canned | 0 1 2 3+ |
|  | Juice or Concentrate | 11.5oz 12oz  48oz  64oz  Other:___ | 0 1 2 3+ |  | Dried | 0 1 2 3+ | Canned | 0 1 2 3+ |
|  | Juice or Concentrate | 11.5oz 12oz  48oz  64oz  Other:___ | 0 1 2 3+ |  | Dried | 0 1 2 3+ | Canned | 0 1 2 3+ |
|  | Juice or Concentrate | 11.5oz 12oz  48oz  64oz  Other:___ | 0 1 2 3+ |  | Dried | 0 1 2 3+ | Canned | 0 1 2 3+ |
| **Total Number of WIC-approved shelf stable Fruit Juice Varieties:** | | **Total Number of shelf stable Fruit Juice Varieties:** |  |  | **Total Number of WIC-approved Dried Fruit Varieties:** | **Total Number of Dried Fruit Varieties:** | **Total Number of WIC-approved Canned Fruit Varieties:** | **Total Number of Canned Fruit Varieties:** |

**Vegetables: Juice and Shelf-Stable**

**Location Juice (Shelf-stable): SD H EL L Other_____________**

**Location (Canned): SD H EL L Other_____________**

**Location (Dried): SD H EL L Other_____________**

| **Vegetables** | **Size (circle one)** | **Amount** | **Shelf-Stable** | **Amount** | **Shelf-Stable** | **Amount** |
| --- | --- | --- | --- | --- | --- | --- |
| **Example: V8 or Carrots** | 48oz. 64oz.  Other:___ | 0 1 2 3+ | Dried | 0 1 2 3+ | Canned | 0 1 2 3+ |
|  | 48oz. 64oz.  Other:___ | 0 1 2 3+ | Dried | 0 1 2 3+ | Canned | 0 1 2 3+ |
|  | 48oz. 64oz.  Other:___ | 0 1 2 3+ | Dried | 0 1 2 3+ | Canned | 0 1 2 3+ |
|  | 48oz. 64oz.  Other:___ | 0 1 2 3+ | Dried | 0 1 2 3+ | Canned | 0 1 2 3+ |
|  | 48oz. 64oz.  Other:___ | 0 1 2 3+ | Dried | 0 1 2 3+ | Canned | 0 1 2 3+ |
|  | 48oz. 64oz.  Other:___ | 0 1 2 3+ | Dried | 0 1 2 3+ | Canned | 0 1 2 3+ |
|  | 48oz. 64oz.  Other:___ | 0 1 2 3+ | Dried | 0 1 2 3+ | Canned | 0 1 2 3+ |
|  | 48oz. 64oz.  Other:___ | 0 1 2 3+ | Dried | 0 1 2 3+ | Canned | 0 1 2 3+ |
| **Total Number of WIC-approved shelf stable Vegetable Juice Varieties:** | **Total Number of shelf stable Vegetable Juice Varieties:** | **Total Number of WIC-approved Dried Vegetable Varieties:** | **Total Number of Dried Vegetable Varieties:** | **Total Number of WIC-approved Canned Vegetable Varieties:** | **Total Number of Canned Vegetable Varieties:** | |

**Whole Grains (SHELF STABLE)**

|  | **Size** | **Amount** | **Price** | **Expired? 1 for Yes** |
| --- | --- | --- | --- | --- |
| **Breakfast Cereals** |  |  |  |  |
| Cheerios | 9oz  12oz  18oz  24oz  36oz | 0  1  2  3+ |  |  |
| Shredded Mini-Wheats | 9oz  12oz  18oz  24oz  36oz | 0  1  2  3+ |  |  |
| Ready-to-Eat Cereal___________________ | 9oz  12oz  18oz  24oz  36oz | 0  1  2  3+ |  |  |
| Ready-to-Eat Cereal___________________ | 9oz  12oz  18oz  24oz  36oz | 0  1  2  3+ |  |  |
| Ready-to-Eat Cereal___________________ | 9oz  12oz  18oz  24oz  36oz | 0  1  2  3+ |  |  |
| Ready-to-Eat Cereal___________________ | 9oz  12oz  18oz  24oz  36oz | 0  1  2  3+ |  |  |
| Ready-to-Eat Cereal___________________ | 9oz  12oz  18oz  24oz  36oz | 0  1  2  3+ |  |  |
| Hot Cereal: Oatmeal | 9oz  12oz  18oz  24oz  36oz | 0  1  2  3+ |  |  |
| Hot Cereal: Grits | 9oz  12oz  18oz  24oz  36oz | 0  1  2  3+ |  |  |
| Hot Cereal: Cream of Wheat | 9oz  12oz  18oz  24oz  36oz | 0  1  2  3+ |  |  |
| **Total Number of WIC-approved Cereal Varieties:** | | **Total Number of Cereal Varieties:** | |  |
| **Tortillas/Breads/Grains** | **Size** | **Amount** | **Price** | **Expired? 1 for Yes** |
| Whole Grain Bread | 160z Other:_____________ | 0  1  2  3+ |  | 0 1 |
| **Total Number of WIC-approved Bread Varieties:** | **Total Number of Bread Varieties:** |  |  |  |
|  | **Size (only Fresh)** | **Amount** | **Price** | **Expired? 1 for Yes** |
| Whole Grain Tortilla: Corn | 160z Other:_____________ | 0  1  2  3+ |  |  |
| Whole Grain Tortilla: Wheat | 160z Other:_____________ | 0  1  2  3+ |  |  |
| Whole Grain Buns | 160z Other:_____________ | 0  1  2  3+ |  |  |
| Whole Grain Rolls | 160z Other:_____________ | 0  1  2  3+ |  |  |
| Whole Grain Pasta | 160z Other:_____________ | 0  1  2  3+ |  |  |
| Brown Rice | 16oz  Other:_____________ | 0  1  2  3+ |  |  |
| Other Whole Grain_________ | 16oz  Other:____________ | 0  1  2  3+ |  |  |
| Other Whole Grain_________ | 16oz  Other:_____________ | 0  1  2  3+ |  |  |
| Other Whole Grain_________ | 16oz  Other:____________ | 0  1  2  3+ |  |  |
| Other Whole Grain_________ | 16oz  Other:___________ | 0  1  2  3+ |  |  |
| **Total Number of WIC-approved Shelf-stable Tortilla Varieties:** | | **Total Number of Shelf-stable Tortilla Varieties:** | | |
| **Total Number of WIC-approved Shelf-stable Roll and Bun Varieties:** | | **Total Number of Shelf-stable Roll and Bun Varieties:** | | |
| **Total Number of WIC-approved Shelf-stable Pasta Varieties:** | | **Total Number of Shelf-stable Pasta Varieties:** | | |
| **Total Number of WIC-approved Shelf-stable Rice Varieties:** | | **Total Number of Shelf-stable Rice Varieties:** | | |
| **Total Number of WIC-approved Shelf-stable Other Grains Varieties:** | | **Total Number of Shelf-stable Other Grains Varieties:** | | |

**Infant Food and Formula**

| **Infant Formula** | **Amount** | **Expired?** | **Price** | **Size** | **Brand(s)** |
| --- | --- | --- | --- | --- | --- |
| Infant formula A (Cow's Milk Based, Concentrated) | 0 1 2 3+ | 0 1 |  |  |  |
| Infant formula A (Cow's Milk Based, Powdered) | 0 1 2 3+ | 0 1 |  |  |  |
| Infant formula A (Cow's Milk Based, Ready-to-Feed) | 0 1 2 3+ | 0 1 |  |  |  |
| Infant formula B (Cow's Milk Based, Concentrated) | 0 1 2 3+ | 0 1 |  |  |  |
| Infant formula B (Cow's Milk Based, Powdered) | 0 1 2 3+ | 0 1 |  |  |  |
| Infant formula B (Cow's Milk Based, Ready-to-Feed) | 0 1 2 3+ | 0 1 |  |  |  |
| Infant formula C (Cow's Milk Based, Concentrated) | 0 1 2 3+ | 0 1 |  |  |  |
| Infant formula C (Cow's Milk Based, Powdered) | 0 1 2 3+ | 0 1 |  |  |  |
| Infant formula C (Cow's Milk Based, Ready-to-Feed) | 0 1 2 3+ | 0 1 |  |  |  |
| Infant formula D (Soy Based, Concentrated) | 0 1 2 3+ | 0 1 |  |  |  |
| Infant formula D (Soy Milk Based, Powdered) | 0 1 2 3+ | 0 1 |  |  |  |
| Infant formula D (Soy Milk Based, Ready-to-Feed) | 0 1 2 3+ | 0 1 |  |  |  |
| Infant formula E (Soy Based, Concentrated) | 0 1 2 3+ | 0 1 |  |  |  |
| Infant formula E (Soy Milk Based, Powdered) | 0 1 2 3+ | 0 1 |  |  |  |
| Infant formula E (Soy Milk Based, Ready-to-Feed) | 0 1 2 3+ | 0 1 |  |  |  |
| ***Exempt Formulas (Hypoallergenic/Specialized/Premature)*** |  |  |  |  |  |
| ___________________(Concentrated) | 0 1 2 3+ | 0 1 |  |  |  |
| ___________________(Powdered) | 0 1 2 3+ | 0 1 |  |  |  |
| ___________________(Ready-to-Feed) | 0 1 2 3+ | 0 1 |  |  |  |
| ___________________(Concentrated) | 0 1 2 3+ | 0 1 |  |  |  |
| ___________________(Powdered) | 0 1 2 3+ | 0 1 |  |  |  |
| ___________________(Ready-to-Feed) | 0 1 2 3+ | 0 1 |  |  |  |
| ___________________(Concentrated) | 0 1 2 3+ | 0 1 |  |  |  |
| ___________________(Powdered) | 0 1 2 3+ | 0 1 |  |  |  |
| ___________________(Ready-to-Feed) | 0 1 2 3+ | 0 1 |  |  |  |
| **Total Number of WIC-approved Infant Formula Varieties:** | | **Total Number of Infant Formula Varieties:** | | | |
| ***Nutritionals*** | **Amount** | **Expired?** | **Price** | **Size** | **Brand(s)** |
| ___________________(Concentrated) | 0 1 2 3+ | 0 1 |  |  |  |
| ___________________(Powdered) | 0 1 2 3+ | 0 1 |  |  |  |
| ___________________(Ready-to-Feed) | 0 1 2 3+ | 0 1 |  |  |  |
| ___________________(Concentrated) | 0 1 2 3+ | 0 1 |  |  |  |
| ___________________(Powdered) | 0 1 2 3+ | 0 1 |  |  |  |
| ___________________(Ready-to-Feed) | 0 1 2 3+ | 0 1 |  |  |  |
| ___________________(Concentrated) | 0 1 2 3+ | 0 1 |  |  |  |
| ___________________(Powdered) | 0 1 2 3+ | 0 1 |  |  |  |
| ___________________(Ready-to-Feed) | 0 1 2 3+ | 0 1 |  |  |  |
| **Total Number of WIC-approved Nutritional Varieties:** | | **Total Number of Nutritional Varieties:** | | | |
| **Infant Cereal** | **Amount** | **Expired?** | **Price** | **Size** | **Brand(s)** |
| Dry Infant Cereal: Oatmeal | 0 1 2 3+ | 0 1 |  |  |  |
| Dry Infant Cereal: Rice | 0 1 2 3+ | 0 1 |  |  |  |
| Dry Infant Cereal: Multigrain | 0 1 2 3+ | 0 1 |  |  |  |
| Dry Infant Cereal: __________________ | 0 1 2 3+ | 0 1 |  |  |  |
| **Total Number of WIC-approved Infant Cereal Varieties:** | | **Total Number of Infant Cereal Varieties:** | | | |
| **Infant Foods** | **Amount** | **Expired?** | **Price** | **Size** | **Brand(s)** |
| Baby Food - Fruits & Vegetables (list single foods first then mixed produce foods, e.g., apples and spinach – do not include mixed meat) | | | | | |
| **Example: Apples** | 0 1 2 3+ | 0 1 |  | 4oz Other:___ |  |
|  | 0 1 2 3+ | 0 1 |  | 4oz Other:___ |  |
|  | 0 1 2 3+ | 0 1 |  | 4oz Other:___ |  |
|  | 0 1 2 3+ | 0 1 |  | 4oz Other:___ |  |
|  | 0 1 2 3+ | 0 1 |  | 4oz Other:___ |  |
|  | 0 1 2 3+ | 0 1 |  | 4oz Other:___ |  |
|  | 0 1 2 3+ | 0 1 |  | 4oz Other:___ |  |
|  | 0 1 2 3+ | 0 1 |  | 4oz Other:___ |  |
|  | 0 1 2 3+ | 0 1 |  | 4oz Other:___ |  |
|  | 0 1 2 3+ | 0 1 |  | 4oz Other:___ |  |
| **Total Number of WIC-approved Baby Food Fruit and Vegetable Varieties:** | | | **Total Number of Baby Food Fruit and Vegetable Varieties:** | | |
| **Baby Food Meats (include mixed meats with fruits/vegetables)** | **Amount** | **Expired?** | **Price** | **Size** | **Brand(s)** |
| Baby Food Meat: ______________________ | 0 1 2 3+ | 0 1 |  | 2.5oz  Other:___ |  |
| Baby Food Meat: ______________________ | 0 1 2 3+ | 0 1 |  | 2.5oz  Other:___ |  |
| Baby Food Meat: ______________________ | 0 1 2 3+ | 0 1 |  | 2.5oz  Other:___ |  |
| Baby Food Meat: ______________________ | 0 1 2 3+ | 0 1 |  | 2.5oz  Other:___ |  |
| Baby Food Meat: ______________________ | 0 1 2 3+ | 0 1 |  | 2.5oz  Other:___ |  |
| Baby Food Meat: ______________________ | 0 1 2 3+ | 0 1 |  | 2.5oz  Other:___ |  |
| **Total Number of WIC-approved Baby Food Meat Varieties:** | | | **Total Number of Baby Food Meat Varieties:** | | |

**Non-food Items**

| **Non-food item sold** | **1 for Yes** | **Brand** | **Size** | **Price** |
| --- | --- | --- | --- | --- |
| **Pots and pans** | **0 1** |  |  |  |
| **Spices** | **0 1** |  |  |  |
| **Cleaning products**  **Laundry detergent (oz)**  **Window cleaner (oz)**  **Disinfecting Wipes (# of wipes/container)** | **0 1**  **0 1**  **0 1** |  |  |  |
| **Toilet paper (units)** | **0 1** |  |  |  |
| **Clothing** | **0 1** |  |  |  |
| **Alcohol** | **0 1** |  |  |  |
| **Tobacco** | **0 1** |  |  |  |

**Shelf Space**

| **Shelf Space** | | |
| --- | --- | --- |
| FOOD CATEGORY | DEFINITION | SHELF SPACE |
| Fresh Fruit | *All fresh fruit* | 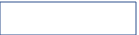feet |
| Fresh Vegetables | *All fresh vegetables* | 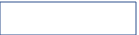feet |
| Frozen Fruit | *All frozen fruit* | 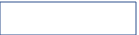feet |
| Frozen Vegetables | *All frozen vegetables* | 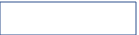feet |
| Canned Fruit | *All canned fruit* | 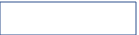feet |
| Canned Vegetables | *All canned vegetables* | 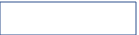feet |
| Bottled water | *INCLUDE only plain water: still & carbonated water with* ***no added caloric/non-caloric sweeteners*** | 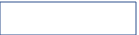feet |
| Sugar sweetened beverages | *INCLUDE all beverages EXCEPT:*   - *Plain water: still & carbonated water with no added caloric/non-caloric sweeteners* - *100% fruit juice, including coconut water* - *Plain milk: cow’s milk and milk substitutes with no added sweeteners* - *Alcohol* | 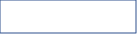feet |
| Packaged Salty Snacks | *INCLUDE all salty snacks EXCEPT:*   - *Nuts and seeds* - *Rice cakes* - *Crackers*   *For example, DO include: chips, popped and/or flavored popcorn, pretzels, salted meat snacks like beef jerky, Cheez-its, Combos, cracker sandwiches and similar salty, processed foods.* | 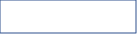feet |
| Packaged Sweet Snacks (Candy & Desserts) | *INCLUDE all sweet snacks EXCEPT:*   - *Refrigerated/ frozen sweet snacks: ice cream and other frozen desserts* - *Cereal and other nutrition bars (e.g., PowerBar, Nutri-Grain bars)* - *Dried fruit*   *For example, DO include: chocolates, other candy, cookies, snack cakes, brownies, pastries/ pies, donuts, sweet rolls and similar sweet processed (shelf stable) foods.* | 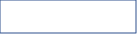feet |

**Appendix C WIC Readiness Score**

| **Categories** |  |  |  |
| --- | --- | --- | --- |
|  | **Score Minimum** | **Score Maximum** | **Proportion Bonus** |
| **Fruit and Vegetables** |  |  |  |
| *Fresh Fruits and Vegetables* |  |  |  |
| Fresh Fruit | 0 | 5 | 2 |
| Fresh Vegetables | 0 | 5 | 2 |
| *Packaged Fruits and Vegetables* |  |  |  |
| Frozen Fruit and Fruit Juice | 0 | 4 | 2 |
| Canned Fruit | 0 | 5 | 2 |
| Dried Fruit | 0 | 5 | 2 |
| Canned Vegetables | 0 | 5 | 2 |
| Dried Vegetables | 0 | 5 | 2 |
| Frozen Vegetables |  | 3 | 2 |
|  |  |  |  |
| **Fruit Juice** |  |  |  |
| Fresh Fruit and Vegetable Juice | 0 | 3 | 2 |
| Shelf-stable Fruit Juice | 0 | 4 | 2 |
| Shelf-stable Vegetable Juice | 0 | 3 | 2 |
|  |  |  |  |
| **Grains** |  |  |  |
| Tortillas | 0 | 1 | 2 |
| Bread | 0 | 1 | 2 |
| Rolls and Buns | 0 | 1 | 2 |
| Pasta | 0 | 1 | 2 |
| Rice | 0 | 1 | 2 |
| Other Grains | 0 | 2 | 2 |
| Cereals (ready-to-eat and hot) | 0 | 4 | 2 |
| Fresh (refrigerated) grains | 0 | 3 | 2 |
| Frozen grains | 0 | 3 | 2 |
|  |  |  |  |
| **Dairy/Dairy Substitutes** |  |  |  |
| Fresh Milk | 0 | 5 | 2 |
| Shelf-stable Milk | 0 | 5 | 2 |
| Cheese | 0 | 5 | 2 |
| Yogurt | 0 | 1 | 2 |
| Lactose Free Milk | 0 | 1 |  |
| Dairy Substitutes (goat and soy milk) | 0 | 1 |  |
| Tofu | 0 | 1 | 2 |
|  |  |  |  |
| **Proteins** |  |  |  |
| Eggs | 0 | 1 | 2 |
| Peanut Butter | 0 | 1 | 2 |
| Canned Fish | 0 | 2 | 2 |
| Beans and Legumes | 0 | 5 | 2 |
|  |  |  |  |
| **Infant Food/Formula** |  |  |  |
| Infant Formula | 0 | 5 | 2 |
| Hypoallergenic/Specialized formula | 0 | 5 |  |
| Nutritional (Toddler Drinks) | 0 | 5 | 2 |
| Infant Cereal | 0 | 5 | 2 |
| Infant Baby Food Fruits and Vegetable | 0 | 5 | 2 |
| Infant Baby Food Meat/Mixed | 0 | 5 | 2 |
|  |  |  |  |
| **Total** | **0** | **122** | **68** |
| **Potential Range** | **0** | **190** |  |

**Appendix D Healthy Food Availability Score**

| **Item Category** | **Sub-category of item** | **Availability of Healthier Item** | **Availability Total Points** | | **Price** | **Price Total Points** | **Quality** | **Quality Total Points** | |
| --- | --- | --- | --- | --- | --- | --- | --- | --- | --- |
|  |  |  | **Min** | **Max** |  |  |  | **Min** | Max |
| Fruits | Fresh | 0 varieties = 0 pts 1-2 varieties =1 pts 3-4 varieties =2 pts < 5 varieties = 3 pts | 0 | 3 |  |  | 25-49% acceptable = 1 pt 50-74% acceptable = 2 pts 75%+ acceptable = 3 pts | 1 | 3 |
|  | Non-fresh | Frozen = 1 Canned = 1 Dried = 1 | 0 | 3 |  |  | N/A |  |  |
| Vegetables | Fresh | 0 varieties = 0 pts 1-2 varieties =1 pts 3-4 varieties =2 pts < 5 varieties = 3 pts | 0 | 3 |  |  | 25-49% acceptable = 1 pt 50-74% acceptable = 2 pts 75%+ acceptable = 3 pts | 1 | 3 |
|  | Non-fresh | Frozen = 1 Canned = 1 Dried = 1 | 0 | 3 |  |  | N/A |  |  |
| Juice | Fresh Fruit juice | 0 varieties = 0 pts 1-2 varieties =1 pts 3-4 varieties =2 pts < 5 varieties = 3 pts | 0 | 3 |  |  | N/A |  |  |
|  | Fresh Vegetable juice | 0 varieties = 0 pts 1-2 varieties =1 pts 3-4 varieties =2 pts < 5 varieties = 3 pts | 0 | 3 |  |  | N/A |  |  |
|  | Shelf-stable Fruit Juice | Frozen (concentrate) =1  1-2 varieties =1 pts 3-4 varieties =2 pts < 5 varieties = 3 pts | 0 | 3 |  |  | N/A |  |  |
|  | Shelf-stable Vegetable Juice | Frozen (concentrate) =1  1-2 varieties =1 pts 3-4 varieties =2 pts < 5 varieties = 3 pts | 0 | 3 |  |  | N/A |  |  |
| Grains and Tortillas (Corn tortilla, Wheat tortilla, Whole grain bread, buns, rolls and pasta)  Cereal | Fresh (comes from a local bakery) | Yes (for any between tortilla/bread/buns/rolls/pasta) = 1  > 2 variety of any = 1 pt | 0 | 2 |  |  | Expired = -1 | -1 |  |
|  | Frozen | Yes (for any between tortilla/bread/buns/rolls/pasta) = 1  > 2 variety of any = 1 pt | 0 | 2 |  |  | Expired = -1 | -1 |  |
|  | Shelf-stable | Yes (for any between tortilla/bread/buns/rolls/pasta) = 1  > 2 variety of any = 1 pt | 0 | 2 |  |  | Expired = -1 | -1 |  |
|  | Cereal | Yes (healthier cereal) = 2pts > 2 varieties of any = 1 pt  Hot cereal = 1 pt | 0 | 4 |  |  | N/A |  |  |
| Dairy | Milk- Fresh | Low-fat/skim milk = 2 pts Whole milk =1 pt  Milk substitute / any plant-based milk substitute = 1 Lactose-free =1 | 0 | 5 | Lower for lowest fat = 2 pts Same for both = 1 pt Higher for low-fat = -1 pt | -1 to 4 | Expired = -1 | -1 |  |
|  | Milk Shelf-stable (Liquid/Powdered) | Yes (for milk/milk substitute/lactose free milk)= 1 pt | 0 | 1 |  |  | Expired = -1 | -1 |  |
|  | Yogurt | Low-fat yogurt = 2 pts Whole milk yogurt =1 pt  Plant-based yogurt = 1 pt | 0 | 4 |  |  | Expired = -1 | -1 |  |
|  | Cheese | Block / Slice cheese / Shredded cheese/Cheese stick =1 pt Plant-based cheese =1 pt | 0 | 2 |  |  | Expired = -1 | -1 |  |
|  | Tofu | Yes= 1 pt | 0 | 1 |  |  | Expired = -1 | -1 |  |
| Proteins | Fresh | Yes (Meat) = 1 pt >2 varieties = 1 pt  Eggs = 1 pt | 0 | 3 |  |  | Expired = -1 | -2 |  |
|  | Frozen | Yes (Meat) = 1 pt >2 varieties = 1 pt  Tofu meal = 1pt | 0 | 3 |  |  | N/A |  |  |
|  | Shelf-stable | Fish (canned) = 1 pt > 2 varieties = 1 pt | 0 | 2 |  |  | N/A |  |  |
|  |  | Beans dried = 1 pt Beans canned =1 pt > 2 varieties (Dry) =1 pt > 2 varieties (Canned) =1 pt  Lentil (D/C) = 1 pt Peas (D/C) = 1 pt | 0 | 6 |  |  | N/A |  |  |
|  |  | Peanut butter Yes = 1 pt | 0 | 1 |  |  | N/A |  |  |
| **Total points** |  |  | **0** | **62** | **-1** | **4** |  | **-10** | **6** |
